# Quantitative MALDI imaging of aspirin metabolites in mouse models of triple-negative breast cancer

**DOI:** 10.7150/thno.116819

**Published:** 2025-08-22

**Authors:** Tae-Hun Hahm, Dalton R. Brown, Caitlin M. Tressler, Thao Tran, Alice Ly, Arvind P. Pathak, Michael T. McMahon, Kristine Glunde

**Affiliations:** 1 The Johns Hopkins Applied Imaging Mass Spectrometry Core and Service Center, Division of Cancer Imaging Research, The Johns Hopkins University School of Medicine, Baltimore, MD 21205, USA.; 2 The Russell H. Morgan Department of Radiology and Radiological Science, The Johns Hopkins University School of Medicine, Baltimore, MD 21205, USA.; 3 Aspect Analytics NV, C-mine 12, 3600 Genk, Belgium.; 4 Department of Biomedical Engineering, The Johns Hopkins University School of Medicine, Baltimore, MD 21205, USA.; 5 The Sidney Kimmel Comprehensive Cancer Center, The Johns Hopkins University School of Medicine, Baltimore, MD 21205, USA Aspect.; 6 F.M. Kirby Research Center for Functional Brain Imaging, Kennedy Krieger Institute, Baltimore, MD 21205, USA.; 7 Department of Biological Chemistry, The Johns Hopkins University School of Medicine, Baltimore, MD 21205, USA.

**Keywords:** aspirin, salicylic acid, MALDI, imaging, quantitative, metabolites

## Abstract

**Rationale:** The non-steroidal anti-inflammatory drug aspirin is currently being developed as activatable contrast agent for chemical exchange saturation transfer (CEST) magnetic resonance imaging (MRI), for detection of its CEST MRI active metabolite salicylic acid (SA). This study refines and develops quantitative matrix-assisted laser desorption/ionization (QMALDI) imaging to investigate the distribution of aspirin metabolites including SA in triple-negative breast cancer (TNBC) models in mice.

**Method:** In this study, we established QMALDI imaging with norharmane (nH) matrix and assisted by the addition of 5 mM peracetic acid (PAA) for optimized SA detection. Deuterated D_6_-SA was added as an internal standard to quantify SA detection. PAA was applied via spraying to improve matrix uniformity and reduce crystal size by forming hydrogen bonds with the nH matrix. Ultraviolet (UV) irradiation during MALDI imaging activated PAA, generating reactive radicals that facilitated the breakdown of nH matrix compounds, thereby reducing matrix-related noise.

**Results:** QMALDI imaging with 5 mM PAA-doped nH matrix and D_6_-SA as internal standard revealed SA accumulation of 141.9 ± 22.6 pmol/mm² in the liver, 129.5 ± 7.8 pmol/mm² in the kidney, and 50.4 ± 3.0 pmol/mm² in TNBC tumors following intravenous injection of aspirin in mice. Precise spatial alignment, integration, and quantification of MALDI imaging, histology, and immunofluorescence images from CD31 staining for blood vessels allowed us to accurately evaluate the spatial distribution of SA in tissue regions enriched with blood vessels and in specific anatomical regions. This spatial data analysis revealed high SA accumulation in the kidney medulla, viable tumor rim containing CD31-stained blood vessels, and throughout the liver.

**Conclusion:** This newly developed QMALDI imaging approach for detecting aspirin metabolites demonstrated high SA accumulation in the kidney medulla and tumor rim containing blood vessels within viable tumor regions following systemic aspirin injection in mice, consistent with our previous study using aspirin-generated SA as activated contrast agent for CEST MRI. This approach enhances the spatial and tissue structural accuracy of quantitative analysis, reinforcing the potential of QMALDI imaging for investigating contrast agents, drug distributions, and metabolism in various tissues.

## Introduction

Aspirin and its metabolite salicylic acid (SA) are both well-established inhibitors of cyclooxygenase 1 and 2 (COX-1 and COX-2), allowing their extensive utilization in treating inflammation by inhibiting prostaglandin synthesis 1. Aspirin is widely used in medicine because of its anti-inflammatory 3, analgesic 4, anti-cancer 5, and antipyretic 6 properties, facilitating the treatment of cardiovascular conditions, stroke, arthritis, and its usage as a chemopreventive agent to prevent cancers, notably breast cancer 1-5. Beyond its therapeutic use, recent studies have identified SA and other salicylates as outstanding contrast agents, particularly for cancer imaging 7-9, through their intramolecular hydrogen bonds which enable strong chemical exchange saturation transfer (CEST) magnetic resonance imaging (MRI) contrast 9-12. As a result, there is renewed interest in the biodistribution of SA and related compounds.

Previously employed methods for measuring the biodistribution of aspirin include autoradiography and gamma scintigraphy which provide precise location information by using 3H, 99mTc-labelled diethylenetriamine pentaacetate (DTPA), samarium-152 oxide, or other forms of radiolabeling 13-15. However, these methods do not provide precise information about the types of metabolites present within tissue, which would be beneficial to know. For example, some side effects caused by aspirin including hearing loss, stomach bleeding, and Reye's syndrome have yet unknown biological mechanisms, which could be revealed if spatial metabolic details were available 16. Furthermore, detoxification of aspirin is thought to occur through glycine conjugation although the literature on the route of this conjugation is limited. A comprehensive understanding of aspirin metabolism and the tissue-specific accumulation of its metabolites would allow for a better understanding of aspirin's side effects, its CEST MRI tumor contrast generation, and its detoxification 17. While CEST MRI can reveal the presence of one or two aspirin metabolites including SA and 2,5-dihydroxybenzoic acid (2,5-DHB) 9, there is an emerging need for new methods which can detect and quantify the presence of all aspirin metabolites in tissue at microscopic spatial resolution.

Our current study utilizes matrix-assisted laser desorption/ionization (MALDI) mass spectrometry (MS) imaging to measure and spatially resolve tissue levels of SA, a metabolite of aspirin as well as a new CEST MRI contrast agent. MALDI imaging is particularly well-suited for the spatially resolved detection of drug metabolites, which inherently exhibit different molecular weights, thereby providing a detailed molecular profile 18. Quantitative MALDI (QMALDI) imaging offers a powerful approach for visualizing and quantifying multiple drug metabolites 19-21. This capability makes QMALDI imaging a valuable tool for drug and contrast agent development studies. In addition, our study introduces an improved matrix spraying technique and an advanced image integration process to enhance the accuracy and precision of QMALDI tissue imaging in conjunction with microscopic slide scanning following histological staining, or immunofluorescence (IF) staining. Developing targeted QMALDI imaging can significantly enhance our understanding of the tissue biodistribution of various drugs, as demonstrated in previous studies 20,21.

The goal of this study was to develop a robust QMALDI imaging technique to investigate the distribution of aspirin metabolites, particularly SA as an activatable CEST MRI contrast agent 9, in organs and tumors from triple-negative breast cancer (TNBC) models in mice which were intravenously injected with aspirin. To investigate the spatial and structural context of the aspirin metabolite distribution in tissues, several microscopic imaging techniques were integrated with QMALDI imaging of SA, which include CD31 IF staining for visualizing vascular networks as well as hematoxylin and eosin (H-E) staining for assessing tissue architecture. The integration of these methodologies establishes a comprehensive framework for visualizing contrast agent distribution, drug metabolism, and tissue characteristics. This approach contributes to the development of enhanced therapeutic strategies and diagnostic applications.

## Methods

**Materials.** Unless otherwise noted, all solvents and trifluoroacetic acid (TFA) were purchased from Sigma Aldrich (St. Louis, MO). Peracetic acid (PAA), norharmane (nH), aspirin (ASA), SA, salicyl acyl glucuronide (SAG), salicyl phenolic glucuronide (SPG), salicyluric acid (SU), 2,5-dihydroxybenzoic acid (2,5-DHB), D-mannitol, glycine, goat serum, Mayer's hematoxylin solution, and aqueous Eosin Y solution were purchased from Sigma-Aldrich (St. Louis, MO, USA). D_6_-salicylic acid (D_6_-SA), 4',6-diamidino-2-phenylindole (DAPI) solution and 4% paraformaldehyde (PFA) were purchased from Thermo Fisher (Waltham, MA). Citrate buffer was purchased from Biolegend (San Diego, CA). All solvents and reagents, which were of analytical grade, were employed without further purification.

**Animal Study and Tissue Collection.** All animal experiments were conducted following protocols approved by the Johns Hopkins University Institutional Animal Care and Use Committee (IACUC). All mice were housed under specific pathogen-free conditions in the Johns Hopkins University animal facility. Two million triple-negative human SUM159 breast cancer cells were inoculated into the right 4th mammary fat pad of 6-8-week-old female athymic nude mice (Envigo RMS LLC, Indianapolis, IN, USA). Tumor-bearing mice with tumor volumes ranging from 300 to 500 mm³ were administered with 100 µL of injectable aspirin (aspirin DL-lysine) at concentrations of 0 mM or 300 mM into their tail veins. Their kidneys, livers, and SUM159 tumors were collected at 60 min following intravenous aspirin administration and frozen in liquid nitrogen vapors. Organs and tumors were stored at -80 °C until MALDI imaging experiments were performed.

**Preparation of Tissue Sections.** Frozen SUM159 tumors, kidneys, and livers were cryo-sectioned into 20-µm-thick sections on a CM1860 UV Cryostat (Leica Biosystems, Wetzlar, Germany) and thaw mounted onto pre-washed, poly-L-lysine coated indium tin oxide (ITO) slides (Delta Technologies, Loveland, CO). These tissue sections were used for quantitative MALDI imaging, as well as H-E and CD31 IF staining analyses, to evaluate and compare their spatial distribution characteristics. The sections were stored in vacuum-sealed slide mailers at -80 °C until matrix deposition. Prior to matrix application, the slides were equilibrated to ambient temperature by placing them in a vacuum desiccator. Norharmane (nH) matrix (10 mg/mL in 70% acetonitrile (ACN) with 0.1% TFA) was prepared with 5 mM peracetic acid (PAA) as an additive and 10 mM D_6_-SA as an internal standard (IS). The solution was uniformly applied to the tissue sections on ITO slides using an HTX M3+ sprayer (HTX Technologies LLC, Chapel Hill, NC). The spraying parameters were set as follows: temperature at 70 °C, nozzle velocity at 1000 mm/min, flow rate at 100 μL/min, with 20 passes and a track spacing of 2 mm. A drying time of 2 s was maintained between passes, and nitrogen gas was supplied at a pressure of 10 psi.

**Polarized Light Microscopy.** Polarized light microscopy images were acquired using an Olympus Slideview VS200 slide scanner (Evident, Waltham, MA, USA) with OlyVIA 4.1.1 software and a 40× objective lens at the Johns Hopkins Applied Imaging Mass Spectrometry (AIMS) Core facility. Linear polarizers were precisely positioned at the front focal plane of the condenser and the rear focal plane of the objective lens. These polarizers were rotated to achieve optimal extinction, with a 95-degree setting used, enabling high-contrast visualization of birefringent materials, such as MALDI matrix crystals.

**Scanning Electron Microscopy (SEM) Analysis.** For SEM analysis, matrix-coated slides with varying concentrations of PAA were mounted on 51 mm holders using copper and carbon adhesive tapes. This ensured stable attachment of the samples and provided the necessary conductivity for electron microscopy. SEM imaging was performed using a JEOL JSM-IT700HR InTouchScope™ microscope (JEOL, Tokyo, Japan) at 3,000× magnification, with an accelerating voltage of 10 kV and a working distance of 13.5 mm. The analysis took place at the Materials Characterization & Processing (MCP) Core Facility. These imaging parameters were optimized to measure high-resolution surface morphology of the matrix-coated samples, allowing for detailed observation of structural features.

**QMALDI Imaging.** The matrix-sprayed tissue sections were analyzed on a timsTOF fleX MALDI-2 mass spectrometer (Bruker Daltonics, Bremen, Germany) equipped with a dual ESI/MALDI source and a 10 kHz smartbeam 3D neodymium (Nd): yttrium Aluminum Garnet (YAG) laser operating at 355 nm for MALDI imaging at the Johns Hopkins Applied Imaging Mass Spectrometry (AIMS) Core facility. Prior to data acquisition, height adjustment (target profile generation) and laser focus tuning were performed. Mass calibration was conducted using electrospray ionization (ESI) of Agilent ESI-L Tune Mix, followed by calibration with red phosphorus to achieve a mass error of less than 1 ppm. MALDI parameters in qTOF mode were optimized to enhance signal intensity by adjusting ion optics, laser intensity, and laser focus. The MALDI acquisition settings included: MALDI plate offset of 50 V, deflection 1 delta of 70 V, funnel 1 RF of 350 Vpp, funnel 2 RF of 350 Vpp, multipole RF of 400 Vpp, collision cell energy of 10 eV, collision RF of 1800 Vpp, quadrupole ion energy of 5 eV with a low mass cutoff at *m/z* 50 Da, focus pre-TOF transfer time of 70 µs, and prepulse storage time of 10 µs. Imaging was performed over the *m/z* range of 50-900 Da in negative ion mode, with raster widths of 100 or 20 µm and 600 shots per pixel at a laser frequency of 10 kHz. Tandem MS experiments were performed with nitrogen as collision gas, using a sum of 5 spectra with 1300 shots per pixel across the *m/z* range of 50-900 Da. The laser power was set to 90% with an isolation window of ±1 Da and a collision energy of 30-40 eV. MALDI imaging data were imported into SCiLS Lab (version 2024b, Bruker Daltonics) for visualization and analysis, focusing on average spectra within the MS imaging region. Average intensity measurements were calculated in SCiLS Lab software by summing all spectra within each region of interest (ROI) and dividing by the total pixel count in that region (standardized at 100 pixels/mm²). Calibration curves were established by analyzing SA standard spots ranging from 2 to 300 pmol/0.2 μL spot. For each standard concentration, circular ROIs (~2 mm² as measured by SCiLS Lab software) were drawn to encompass the entire dried droplet spot area. The ratio of SA signal intensity to D_6_-SA (internal standard) intensity was calculated for each concentration and plotted to generate the calibration curve. The weight of the tissue section (20 μm) was 343 μg/liver section, 356 μg/kidney section, and 450 μg/SUM159 tumor section. Tissue section areas were determined by pixel count analysis in SCiLS Lab software, equating to 48.84 ± 1.66 mm² for liver sections (4884 ± 166 pixels), 44.03 ± 0.63 mm² for kidney sections (4403 ± 63 pixels), and 34.76 ± 0.76 mm² for SUM159 tumor sections (3476 ± 77 pixels). The final SA tissue concentration was expressed as pmol/mm², calculated by applying the calibration curve equation to the measured SA/D_6_-SA intensity ratios from each tissue section, enabling direct comparison of SA accumulation across different tissue types. Limits of detection (LOD) and quantification (LOQ) were calculated based on previously established equations 22. Tandem MS spectra were processed and analyzed using dataAnalysis (version 5.3.236, Bruker Daltonics).

**MALDI-MS Analysis for Validating the PAA Mechanism.** Frozen tissue sections (liver, kidney, and SUM159 tumor) were prepared as described previously using 20-μm-thick cryosections mounted on ITO slides. Each tissue type was sectioned to provide three sequential sections for the three experimental conditions to ensure comparable tissue morphology across treatments. Matrix solutions were applied using an HTX M3+ sprayer under identical conditions: temperature at 70°C, nozzle velocity at 1000 mm/min, flow rate at 100 μL/min, with 20 passes and a track spacing of 2 mm. A drying time of 2 s was maintained between passes, and nitrogen gas was supplied at 10 psi pressure. Three matrix conditions were prepared to test the role of hydroxyl radicals in the PAA mechanism as follows. Control: nH matrix solution (10 mg/mL in 70% ACN with 0.1% TFA) without additives; PAA: nH matrix solution with 5 mM PAA; PAA + scavenger: nH matrix solution with 5 mM PAA and 100 mM D-mannitol. MALDI imaging was performed on a timsTOF fleX MALDI-2 mass spectrometer using identical acquisition parameters across all three conditions. Key analytical targets included the matrix background signals at *m/z* 154.97 and *m/z* 248.96 (nH matrix-derived ions), the hydroxyl radical scavenger D-mannitol at *m/z* 181.07 ([M-H]⁻ ion of D-mannitol). Imaging parameters were set to 100 μm pixel size in negative ion mode, with 600 shots per pixel at 10 kHz laser frequency. MALDI imaging data were processed using SCiLS Lab software (version 2024b, Bruker Daltonics). ROI analysis was performed to observe signal intensities of matrix background ions (*m/z* 154.97 and 248.96) across the three experimental conditions. Average spectra were generated from equivalent tissue regions to compare signal intensity changes.

**Immunofluorescence Staining and Microscopy**. Following MALDI imaging, SUM159 tumor sections were immersed in 100% ethanol for a minimum of 24 h to remove the MALDI matrix. The sections were then rinsed with ethanol and fixed in freshly prepared 4% paraformaldehyde (PFA) at room temperature for 15 min. Post-fixation the tumor sections were incubated in a 10 mM citrate buffer (pH 6.0) followed by a 0.01 M phosphate-buffered saline (PBS, pH 7.2) wash. Subsequently, the sections were blocked in a solution containing 20% goat serum, 1% bovine serum albumin (BSA), 0.3 M glycine, and 0.01 M PBS (pH 7.2) for 24 h at 4 °C. The sections were then incubated with a primary anti-CD31 antibody (1:20, R&D Systems, Minneapolis, MN) for 24 h at 4 °C. CD31 immunostaining was completed by incubating the sections with donkey anti-goat IgG (H+L) highly cross-adsorbed secondary antibody, Alexa Fluor™ Plus 647 (1:100, Invitrogen, Carlsbad, CA) at 37 °C for 3 h. Nuclei were stained with DAPI (1:1000) for 2 min, then washed with PBS for 5 min at 70 rpm shaking before mounting. Imaging of DAPI and CD31 to visualize mouse tumor vasculature was performed using an Olympus Slideview VS200 slide scanner equipped with OlyVIA 4.1.1 software and a 40× objective lens at the Johns Hopkins Applied Imaging Mass Spectrometry (AIMS) Core facility. The imaging was conducted with excitation wavelengths of 378/52 nm for DAPI (emission at 432/36 nm) and 635/18 nm for Alexa 647 (emission at 652/42 nm), respectively.

**Histological Staining.** Hematoxylin and eosin (H-E) staining was performed on the same sections used for MALDI imaging experiments. The residual matrix was removed by submerging slides in 100% ethanol (24 h). Slides were submerged in 70% ethanol for a second time (3 min), followed by Milli-Q water (3 min). Staining was performed in hematoxylin (3 min), followed by rinsing under running tap water (3 min), eosin (30 s), running tap water (1 min), 100% ethanol (1 min), and xylene (30 s). Optical images were acquired at 40× magnification using an Olympus Slideview VS200 slide scanner equipped with OlyVIA 4.1.1 software (Evident, Waltham, MA, USA).

**Integrative Data Analysis Approach using the Weave Software.** All the following image analysis steps were conducted using the new Weave Platform-Version v1.217.11 (Aspect Analytics NV, Genk, Belgium). The WEAVE software integrates MALDI-MS imaging (MSI) with various optical images by image co-registration and resampling of the MALDI-MSI pixels (20 µm/pixel) with nearest neighbor interpolation to exactly match the dimension and pixel spacing of immunofluorescence (IF) and H-E microscopy images (0.325 µm/pixel). In effect, this means there are multiple IF or H-E pixels underneath each MSI pixel after transformation. Mean value aggregation of multiple IF or H-E pixels that are underneath a single MSI pixel is then possible.

For preprocessing, raw MALDI imaging data were converted to imzML format for further processing. The imaging data underwent preprocessing with total ion current (TIC) normalization to standardize signal intensities across all spectra. We selected peaks corresponding to SA, defined by their respective *m/z* values of 137.0247 Da. To ensure accurate peak detection, intensities were aggregated using a tolerance window of ±0.05 Da around these *m/z* values. The binned intensities were then utilized for generating ion images, facilitating subsequent data integration and visualization.

For integrating microscopic imaging modalities, H-E, MALDI, and IF images were manually co-registered using non-rigid registration 23. To align the MALDI imaging data with the corresponding H-E image, ion images of SA were utilized directly to identify landmarks for the non-rigid image co-registration. Similarly, the IF image was co-registered to the H-E image using the same manual non-rigid workflow in the Weave Platform. Finally, both MALDI ion images and IF images were transformed into the spatial coordinate system of the corresponding H-E images.

After aligning the MALDI ion and IF images, IF image pixels (0.325 µm/pixel) were down sampled to the resolution of the MALDI ion image pixel masks (20 µm/pixel) for the calculation of mean IF intensity values per MALDI ion image pixel. This approach allowed us to perform quantitative comparisons of MALDI ion image signals in regions with varying levels of fluorescence staining intensity.

The resulting integrated dataset, comprising MALDI ion image intensities and mean IF intensities for each MALDI ion image pixel, enabled further analysis and extraction of MALDI ion image signal intensities based on fluorescence intensity thresholds in the CD31 stained IF image. Finally, the fused H-E, MALDI-MSI, and IF imaging data stacks were visualized using reports generated in the Weave Platform.

For tumor and kidney tissues, H-E-stained images with annotated regions were co-registered with MALDI images of SA using SCiLS Lab software. The normalized SA intensity was calculated for each defined region from these co-registered images. This assessment was conducted by measuring the SA intensity per pixel across all samples, and data visualization with violin plots was accomplished in R (version 4.4.3) using multiple graphical packages including lattice, plot3D, vioplot, and RColorBrewer. The comparative analysis was presented as an enhanced 3D violin plot incorporating multiple layers of visual information.

**Statistical Analysis.** Statistical analysis was implemented in R (version 4.4.3) utilizing packages including dplyr and base R statistical functions. Raw data were imported from a CSV file containing normalized SA intensity values for two regions. Following data preprocessing to remove "Not Applicable" values, descriptive statistics (median, mean, minimum, maximum, first and third quartiles, and standard deviation) were calculated for each tissue region. To determine if pixel-wise intensity distributions were significantly different between two ROIs from the same tissue section, a two-tailed Student's t-test was performed using R (version 4.4.3) and JSAP 0.19.3 (JSAP Software, Netherlands).

## Results and Discussion

### Peracetic Acid as Spray Additive for MALDI Tissue Imaging of Aspirin Metabolites

When optimizing MALDI matrices for qualitative and quantitative MALDI imaging, the interference caused by noise signals originating from matrix, metals, and other impurities in the tissue sample during the MALDI desorption and ionization process poses a challenge 24. This is particularly relevant for aspirin metabolites because there is overlap between matrix ions and aspirin metabolites in the low mass range (*m/z* 0-500 Da) 21. These noise signals can obscure the signals of interest, making it difficult to obtain a clear and accurate profile of drug distribution and metabolism. Therefore, removing matrix noise is an essential procedure and challenge for both qualitative and quantitative MALDI imaging analysis. In this study, we employed the nH matrix, which was previously used for detecting SA at *m/z* 137.02 Da in negative ion mode by MALDI profiling analysis 9. Additionally, in this study, we tested for the first time the addition of low concentration peracetic acid (PAA) to nH matrix to further enhance the MALDI imaging data, which capitalizes on PAAs ability to generate reactive radicals when exposed to ultraviolet (UV) light. Upon UV irradiation, the bond between oxygen atoms in PAA breaks, forming reactive species as follows: CH_3_CO_3_H 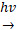
CH_3_CO^•^_2_ + **^•^**OH 25,26. We hypothesized that the generation of these radicals will enhance PAA's oxidative reactivity during MALDI imaging, which uses UV light from a focused laser beam (e.g., a Nd: YAG laser at 355 nm), to break down nH matrix-related ions and remove matrix noise, as the nH matrix has a similar chemical structure as diclofenac and carbamazepine, which are reportedly broken down by PAA 27.

**Figure 1:**
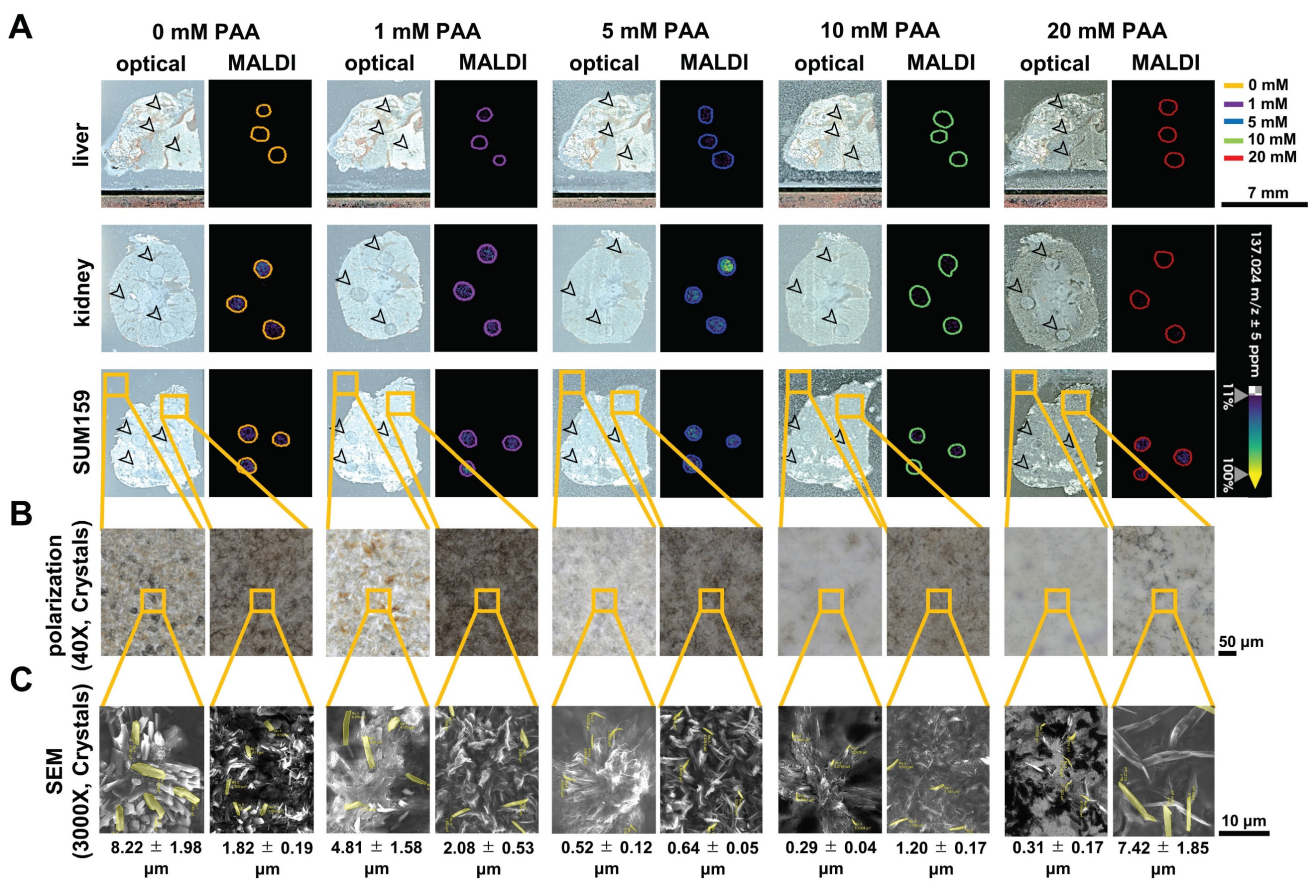
** Effect of PAA concentration on SA detection in tissue samples.** (**A**) Optical and MALDI images of tissue sections spotted with dried droplets of 1 mM salicylic acid (SA) prior to application of norharmane (nH), combined with varying concentrations of peracetic acid (PAA) (0 mM, 1 mM, 5 mM, 10 mM, and 20 mM). Black arrows indicate the locations of the dried SA droplets in the optical images. (**B**) Polarized light microscopy (40×) and (**C**) scanning electron microscopy (SEM, 3000×) images were taken from the orange square-marked regions shown in panels A and B. Matrix-Assisted Laser Desorption/Ionization (MALDI) images were acquired at a 100 µm pixel resolution in negative ion mode using a timsTOF fleX MALDI-2 instrument. The data highlight the detection of the [M-H]⁻ ion of SA at *m/z* 137.02 Da in dried droplets spotted onto tissue sections. The average SEM crystal size was determined from five randomly selected crystals (highlighted in yellow) in each image and is presented as the mean ± standard deviation (SD).

To investigate changes in SA intensity in MALDI imaging, 1 mM SA was applied as dried droplets on tissue sections from control mice (not treated with aspirin) prior to spraying the tissues with nH matrix. Then, nH matrix was sprayed onto these tissue sections with varying concentrations of PAA (0, 1, 5, 10, and 20 mM) as an additive. An increase in SA intensity was observed when adding 1 mM PAA as compared to control (0 mM PAA) (Figure 1 and Table 1). Furthermore, the SA intensity in tissue sections sprayed with nH with the addition of 5 mM PAA showed a substantial increase across various tissues, including liver, kidney, and SUM159 tumor, however, no significant changes in SA intensity were detected at higher PAA concentrations (10 mM and 20 mM). This effect was particularly pronounced in liver tissue, where a more than three-fold increase in the average SA signal intensity was observed, resulting in a significant enhancement in SA detection with 5 mM PAA as spray additive. Furthermore, to address concerns regarding the fragmentation of SA and its potential degradation by 5 mM PAA, we conducted a targeted analysis focusing on the known SA fragment ion at *m/z* 93.03 Da. As demonstrated in Figure S1, this specific ion was not detected in dried droplet samples on tissue sections following PAA application. The absence of this fragment provides strong evidence that SA remains stable under our experimental conditions, with no significant degradation occurring due to PAA treatment.

There were additional benefits as well. At a concentration of 5 mM PAA, the PAA and nH matrix demonstrated a more uniform distribution of co-crystals, as observed in polarization images taken at 40× magnification, compared to samples sprayed with other PAA concentrations (Figures 1B and 1C). The SEM images of the nH matrix crystals with 5 mM PAA revealed significantly thinner and smaller crystal sizes (0.64 ± 0.05 μm²) than those formed at 0, 1, 10, and 20 mM PAA concentrations on tissue sections. The increased crystal uniformity and smaller crystal sizes achieved with 5 mM PAA are crucial for ensuring reproducible matrix application, which is essential for QMALDI imaging analysis 20. As previously reported, hydrogen bonding interactions between the MALDI matrix and additives could lead to the formation of compact crystal structures 20. Strong N-H···O hydrogen bonds contribute to tighter packing and reduced matrix crystal size 28,29. The concentration of the additive plays a crucial role in co-crystallization, affecting both crystal size and uniformity, which is essential for achieving reproducible and effective MALDI analysis 20,30. In this study, the nitrogen atom in the pyridine ring of the nH matrix acts as a hydrogen bond donor, while the carbonyl oxygen (C=O) from PAA serves as a hydrogen bond acceptor. Consequently, hydrogen bonds can form between the carbonyl group of PAA and the N-H group from the nH matrix. The optimal concentration for co-crystallization with the nH matrix was identified as 5 mM PAA (Figure 1), resulting in smaller, evenly distributed crystals which help prevent inhomogeneities and reduce the risk of analyte delocalization, a common issue associated with larger crystals that can lower the resolution of MALDI-MSI 30,31. When the PAA concentration exceeded 10 mM, crystal formation on tissue became less consistent, resulting in larger crystals compared to those formed off-tissue, which negatively impacted the signal intensity of SA in MALDI imaging.

The addition of 5 mM PAA in the spray enhanced the signal intensity of SA in the tissues of SUM159 tumors, livers, and kidneys of aspirin-treated mice without causing SA delocalization, while reducing noise signals from the matrix both on and off tissue (Figures S2, S3, and S4). Notably, MALDI images showed high background noise signals at *m/z* 154.97 Da and *m/z* 248.96 Da, which were significantly reduced after spraying of nH with 5 mM PAA as additive. The background signals at *m/z* 154.97 Da and *m/z* 248.96 Da were identified as originating from the nH matrix, rather than from endogenous tissue components (Figures S5 and S6). This was confirmed by comparing the fragmentation patterns of *m/z* 154.97 Da and *m/z* 248.96 Da between dried nH droplets and tissue sections from liver, kidney, and SUM159 tumor samples using tandem mass spectrometry. To directly validate our proposed mechanism that PAA reduces matrix background through MALDI imaging UV laser-induced hydroxyl radical generation, we conducted radical scavenging experiments using D-mannitol as a specific hydroxyl radical scavenger 32. Three experimental conditions were compared: Control (nH matrix only), PAA (nH matrix + 5 mM PAA), and PAA + scavenger (nH matrix + 5 mM PAA + 100 mM D-mannitol) (Figure S7). MALDI imaging analysis of matrix background signals demonstrated clear evidence supporting the hydroxyl radical mechanism. The characteristic nH matrix-derived ions at *m/z* 154.97 (Figure S7B) and *m/z* 248.96 (Figure S7C) showed reduced signal intensity following addition of PAA compared to control, and importantly, restoration of signal intensity was observed with PAA + scavenger where D-mannitol (Figure S7D) was present. This signal restoration was observed consistently across liver, kidney, and SUM159 tumor tissues. The combined effects of the MALDI imaging UV laser in conjunction with 5 mM PAA additive sprayed together with the nH matrix effectively reduced matrix noise signals without compromising the ionization of SA, the primary drug metabolite in this study.

Overall, the addition of 5 mM PAA significantly enhanced the SA peak intensity across all tested tissues. Compared to controls without PAA, we observed fold increases of 3.2 in liver, 1.8 in kidney, and 1.7 in SUM159 tumor tissues, resulting in a significant improvement in MALDI imaging of SA. The PAA spray additive for nH matrix deposition also improved matrix uniformity and led to a reduction in matrix crystal size, likely due to hydrogen bonding between PAA and nH matrix. However, when the concentration of PAA was increased to greater than 10 mM, both the matrix and dried SA droplets on the tissue underwent degradation, resulting in a decrease in SA intensity (Figure 1 and Table 1). This high PAA concentration also led to the formation of larger, uneven crystals on the tissue, which likely interfered with MALDI imaging 20,30,31. Therefore, our study clearly supports maintaining the PAA concentration at 5 mM to avoid these undesirable effects.

### PAA-Assisted MALDI Imaging of the Distribution of Aspirin Metabolites in Various Tissues Sections

**Figure 2:**
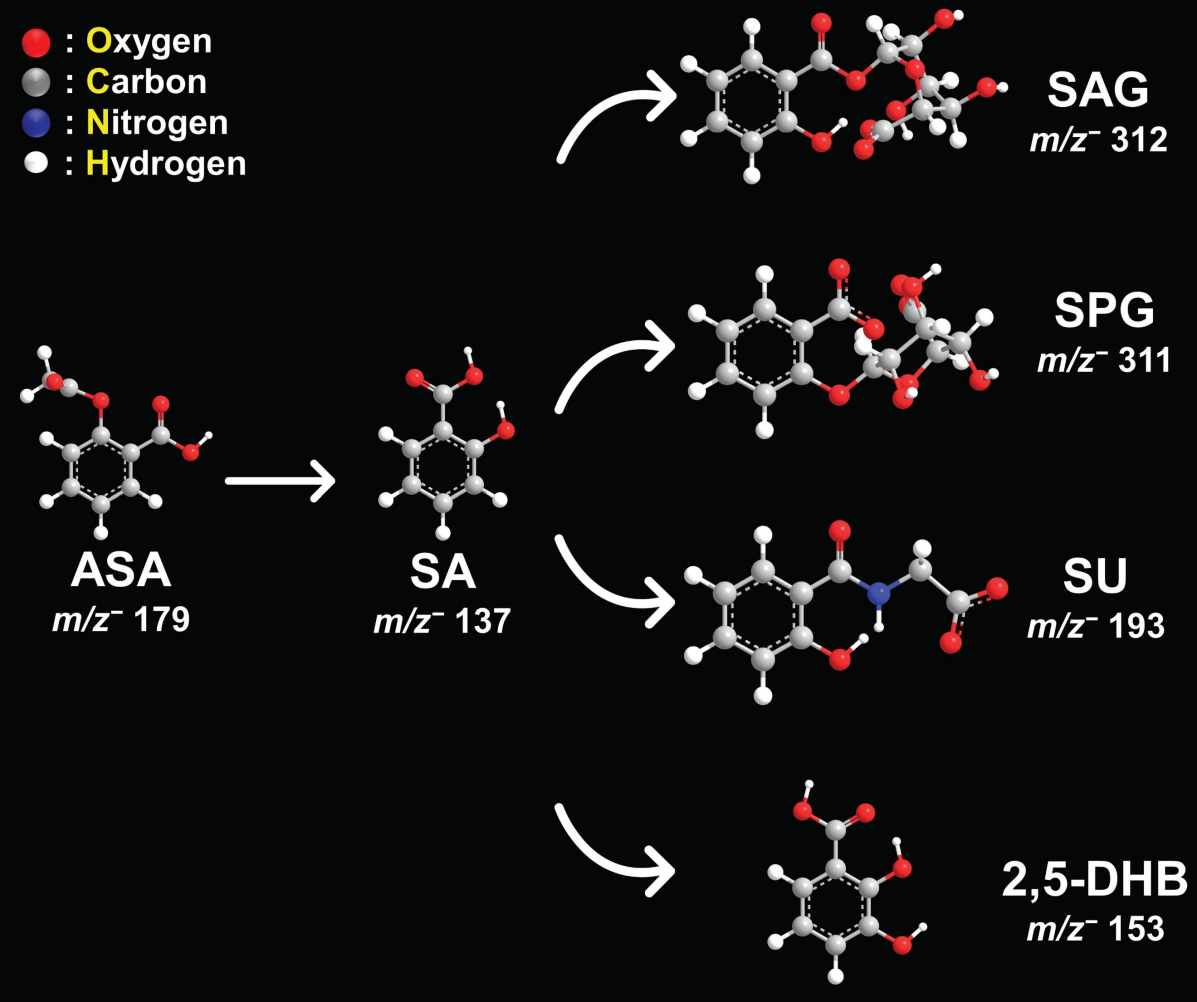
**Schematic representation of aspirin metabolites.** Chemical structures and *m/z* values in negative ion mode for aspirin (ASA), salicylic acid (SA), salicyl acyl glucuronide (SAG), salicyl phenolic glucuronide (SPG), salicyluric acid (SU), and 2,5-dihydroxybenzoic acid (2,5-DHB).

**Figure 3:**
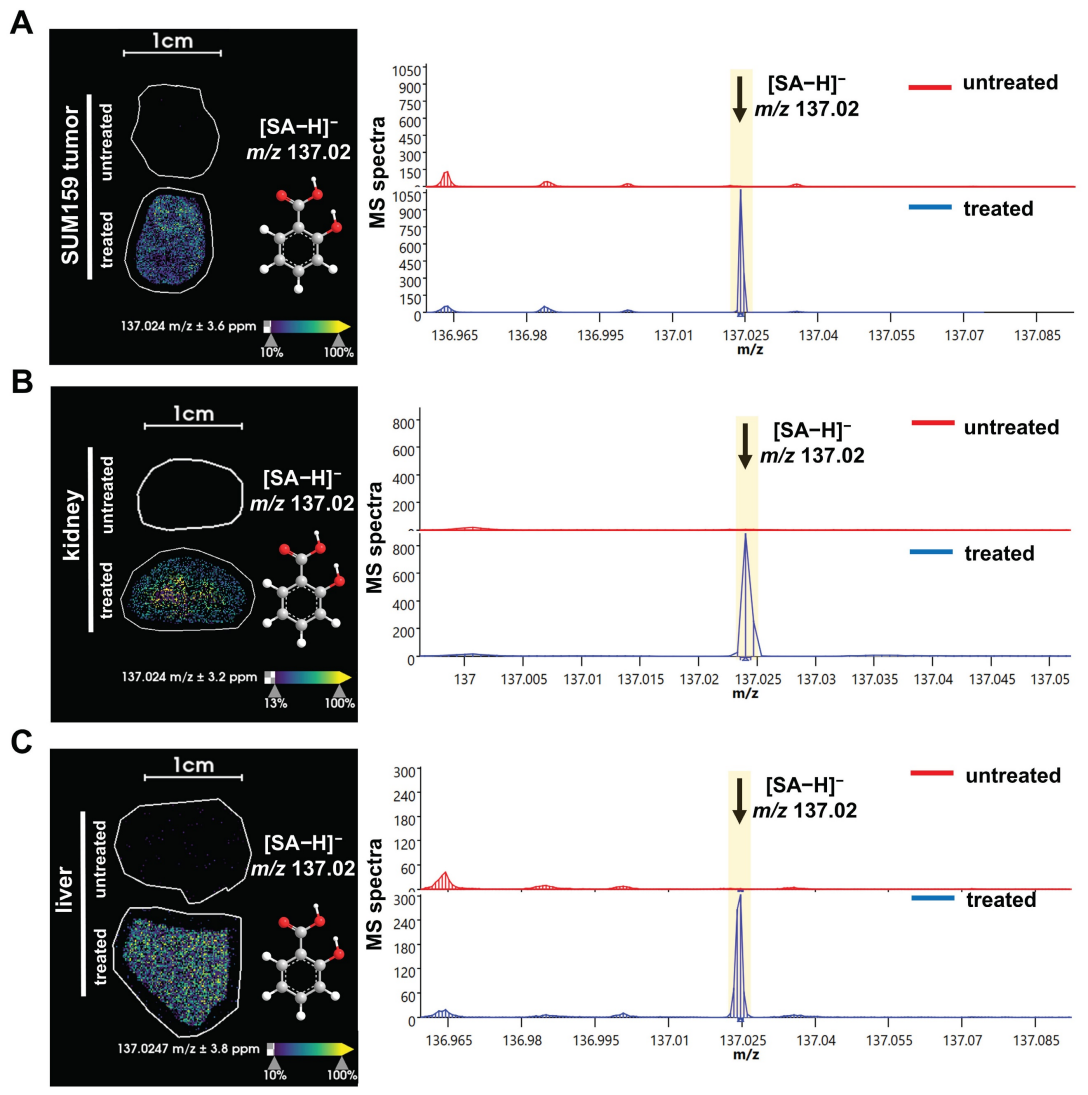
**MALDI imaging of SA distribution in tissues from aspirin treated versus untreated mice.** MALDI images and corresponding average spectra of SA distribution in (**A**) liver, (**B**) kidney, and (**C**) orthotopic SUM159 tumor xenograft of representative central tissue sections obtained from untreated control mice (top, red spectra) versus treated, 300 mM aspirin-injected mice (bottom, blue spectra). The data clearly show that the [M-H]⁻ ion of SA at *m/z* 137.02 Da is detected in tissue sections from treated mice, while it is absent in tissues from untreated mice. The MALDI images were acquired at 100 µm pixel size in negative ion mode using a timsTOF fleX MALDI-2 instrument using norharmane matrix and peracetic acid additive.

Next, we used this new approach to study the aspirin and SA distributions within various tissues. The structures and masses of aspirin, SA, and several other aspirin metabolites are shown in Figure 2. We examined the spatial distribution of the CEST MRI contrast active metabolite SA in tissues from mice growing SUM159 TNBC xenografts in their mammary fat pads, which were systemically injected into their tail veins with 300 mM aspirin and compared these mice to untreated control mice. SA was detected in the liver, kidney, and tumor tissues from SUM159 TNBC bearing mice treated with 300 mM aspirin but not in tissues from untreated control mice (Figure 3). To further validate the detection of SA in tissue sections following aspirin treatment, MALDI imaging was coupled with on-tissue tandem mass spectrometry. This analysis successfully identified a diagnostic SA fragment ion at *m/z* 93.03 Da, which was generated by fragmenting the molecular SA ion of [M-H]⁻ at *m/z* 137.02 Da, confirming the presence of SA in TNBC tumors, as well as in livers and kidneys from aspirin-injected mice (Figure S13). Aspirin was identified in the kidneys as well, confirmed by the detection of its diagnostic fragment ion at *m/z* 137.03 Da from fragmenting the molecular aspirin ion of [M-H]⁻ at *m/z* 179.03 Da (Figures S8 and S14). While there was a small peak detected at *m/z* 179.03 Da in the aspirin-treated livers, it was not significantly above the background that was detected in untreated control mice. Additionally, 2,5-DHB was detected in TNBC tumors, as well as in liver and kidney tissues. The identification of 2,5-DHB was supported by the diagnostic fragment ion at *m/z* 109.03 Da, which was generated by fragmenting the molecular 2,5-DHB ion of [M-H]⁻ at *m/z* 153.02 Da (Figures S9 and S15). However, salicyl acyl glucuronide (SAG) at *m/z* 312.04 Da, salicyl phenolic glucuronide (SPG) at *m/z* 311.04 Da, and salicyluric acid (SU) at *m/z* 193.03 Da were not detected in any of the tissues analyzed, as illustrated in Figures S10, S11, and S12. This may result from low tissue metabolite levels of SAG, SPG, and SU below the detection threshold of MALDI imaging, a reduced ionization efficiency of SAG, SPG, and SU with MALDI, or more rapid breakdown, or less tissue accumulation of SAG, SPG, and SU as compared to aspirin, SA, and 2,5-DHB. Taken together, these results provide valuable information on the spatial distribution of several aspirin metabolites in various tissues and will allow us to further investigate and deepen our knowledge of the use of aspirin in cancer therapy and imaging.

### QMALDI Imaging of SA in Tissue Sections with PAA Spray Additive and Deuterated Internal Standard

We further developed QMALDI imaging of SA by using PAA-assisted spraying of nH matrix and deuterated SA (D_6_-SA) as internal concentration standard to allow us to measure the absolute amount of SA in various tissues. Previous studies have demonstrated that the use of an internal standard is critical for accurate quantification in QMALDI imaging as it compensates for potential variability in ionization efficiency across different tissue types and tissue regions 20,33. This is necessary because MALDI imaging can be affected by tissue composition, potentially leading to variations in signal intensity for the same analyte in different tissues and tissue regions because of differences in the molecular environment and local ion suppression 20,33,34. By incorporating a deuterated internal standard, such tissue regional variations can be effectively normalized for 33, allowing for reliable and reproducible quantification of the target analyte across different tissue sections and regions.

**Figure 4:**
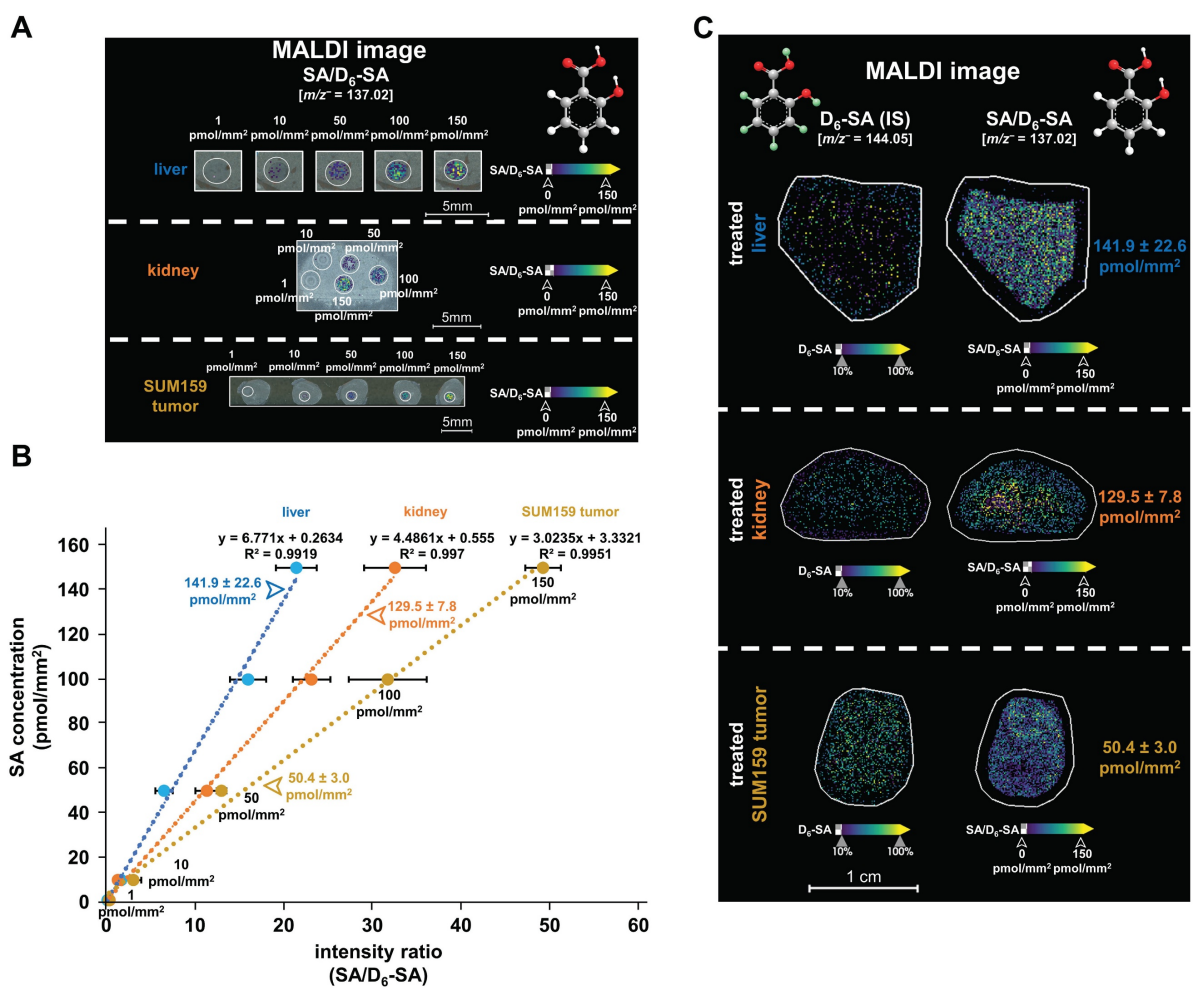
**Quantification of SA in liver, kidney, and SUM159 tumor tissue sections.** (**A**) MALDI images of salicylic acid dried droplets (SA, [M-H]⁻, *m/z* 137.02 Da, 0.5-150 pmol/mm²) were spotted onto untreated tissue sections, measured by QMALDI imaging in negative ion mode, and are shown as normalized SA images of the SA-to-D**_6_**-SA ratio. (**B**) Calibration curves of SA were generated using QMALDI imaging in negative ion mode with D**_6_**-SA as deuterated internal standard and peracetic acid as additive. SA concentration plotted versus SA-to-D_6_-SA intensity ratios generated calibration curves. MS intensities were derived from the average spectra of each circled SA dried droplet spot for each SA concentration as shown in panel A. (**C**) MALDI images of D**_6_**-SA and normalized images of SA-to-D**_6_**-SA ratio were acquired and are shown from data analysis in SCiLS lab. The measured concentrations of SA in the liver, kidney, and SUM159 tumor tissues of mice injected with 300 mM aspirin were 141.9 ± 22.6 pmol/mm², 129.5 ± 7.8 pmol/mm², and 50.4 ± 3.0 pmol/mm², respectively, as shown in panels B and C. The results are presented as mean ± SD from three independent technical replicates of tissues.

Following this quantification approach (Figure S16), the MALDI images in Figures 4A show our calibration curves from dried droplet spots containing various SA concentrations on liver, kidney, and SUM159 tumor sections. Figure 4B shows the resulting linear correlations between the relative signal intensities of SA dried droplets (ranging from 1 to 150 pmol/mm²) normalized to D_6_-SA in liver (R² = 0.9919), kidney (R² = 0.997), and SUM159 TNBC tumor (R² = 0.9951) tissue sections prepared with 5 mM PAA, D_6_-SA, and nH matrix. Figure 4C demonstrates the uniform spatial distribution of D_6_-SA, which is used as a deuterated internal standard, across liver, kidney, and SUM159 TNBC tumor tissue sections following the application of the D_6_-SA spray, visualized by MALDI imaging. D_6_-SA was selected as the internal standard due to the enhanced stability of deuterated compounds under oxidative conditions compared to their non-deuterated counterparts 35,36. The use of D_6_-SA enabled the normalization of both SA intensity and its spatial distribution within the tissue sections, as shown in Figures 4A, 4C, and S17. This approach effectively compensated for variations in ionization efficiency across different tissue types and regions, allowing for quantitative comparison of ion images on an SA/D_6_-SA intensity ratio scale, thereby ensuring accurate and reliable quantification of SA.

SA concentrations were quantitatively measured in liver, kidney, and SUM159 TNBC tumor tissues using three technical replicates per tissue type, where QMALDI imaging measurements were averaged from three directly adjacent tissues to enhance accuracy. The resulting SA tissue concentrations were 141.9 ± 22.6 pmol/mm² in the liver, 129.5 ± 7.8 pmol/mm² in the kidney, and 50.4 ± 3.0 pmol/mm² in the SUM159 TNBC tumor for three technical replicates per tissue type (Table 2). Additionally, biological replicates with three tissue samples each from three different mice were measured (Figures 6A, 7A, and S18) and showed an SA tissue concentration of 133.0 ± 44.6 pmol/mm² in the liver, 140.3 ± 23.3 pmol/mm² in the kidney, and 34.5 ± 10.8 pmol/mm² in the SUM159 TNBC tumor as quantified from three biological replicates each (Table 2). These results demonstrate that liver and kidney tissues contained higher SA tissue concentrations than SUM159 TNBC tumors.

Aspirin is known to rapidly hydrolyze into salicylic acid (SA) once it enters the body 37,38. Therefore, it is not surprising that SA was detected and quantified in all tissues at 60 minutes following administration in this study. When aspirin is taken orally, it passes through the stomach wall and is metabolized in the liver before reaching other tissues 37. However, in our experiments, we used intravenous administration, which allowed aspirin to directly enter the bloodstream, bypassing the stomach and liver, resulting in faster delivery to the tumor compared to oral administration. This likely minimized conversion into various metabolites, enabling more of the drug to reach the target organs and tumor. At 60 minutes post-injection, it is possible that SA concentrations had already peaked in various organs and entered the excretion phase, or that different tissues accumulated SA at different rates. However, since this study only analyzed tissues at the 60-minute time point, we could not observe concentration changes over time or measure SA levels in the blood. Future studies incorporating time-dependent tissue sampling and blood concentration analysis will be essential for a more comprehensive understanding of SA's spatial distribution and pharmacokinetics. Additionally, we observed larger standard deviations in SA tissue concentration between biological repeats compared to technical replicates (Table 2), which shows that the biological variability was larger than the technical variability. The high consistency observed between technical as well as biological replicates reinforces the reliability of the newly developed QMALDI imaging technique for detecting the spatial distribution and absolute quantitation of SA in tissue sections. These findings demonstrate that this technique provides accurate, quantitative spatial analysis of SA distributions in various tissue sections, highlighting its capability as a robust approach for studying drug metabolites in complex tissue samples.

### Integrated Spatial Analysis of QMALDI SA Imaging with H-E Staining of Tissue Architecture and CD31 Immunofluorescence Staining of Vasculature

**Figure 5:**
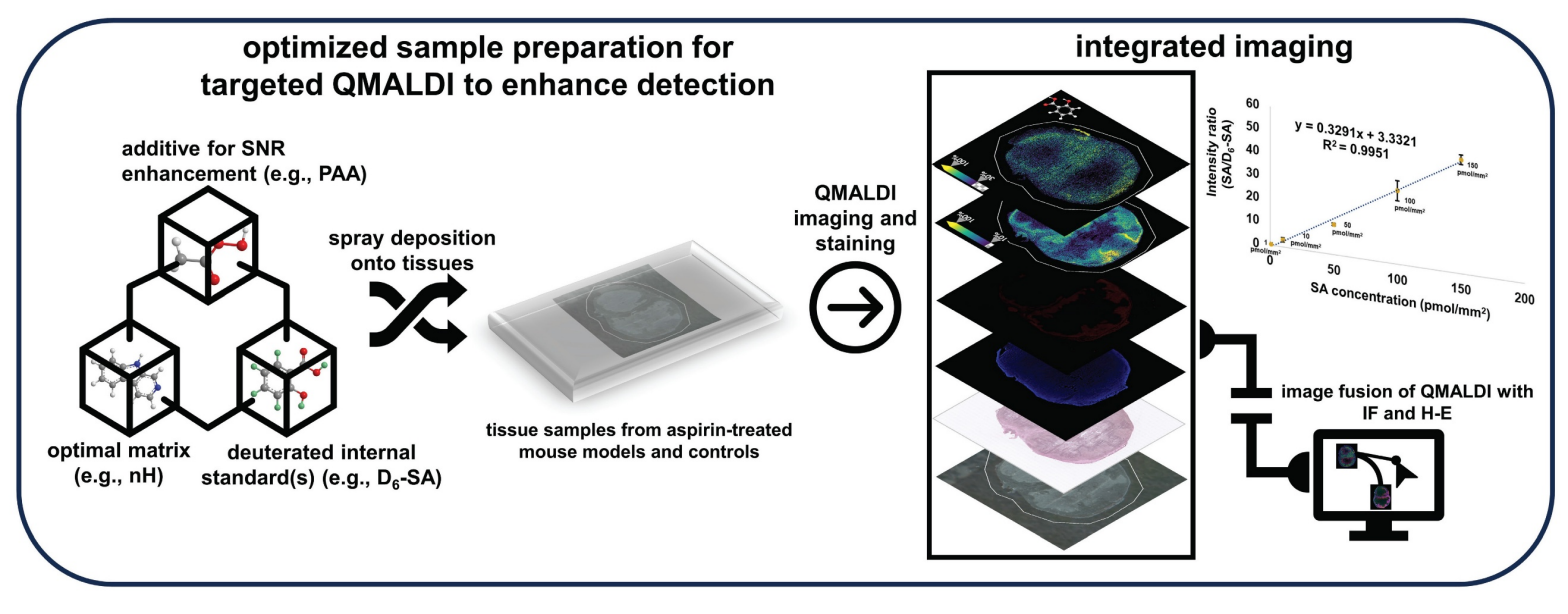
Schematic illustration of the integration of QMALDI imaging with hematoxylin and eosin (H-E) and CD31 immunofluorescence (IF) staining.

**Figure 6:**
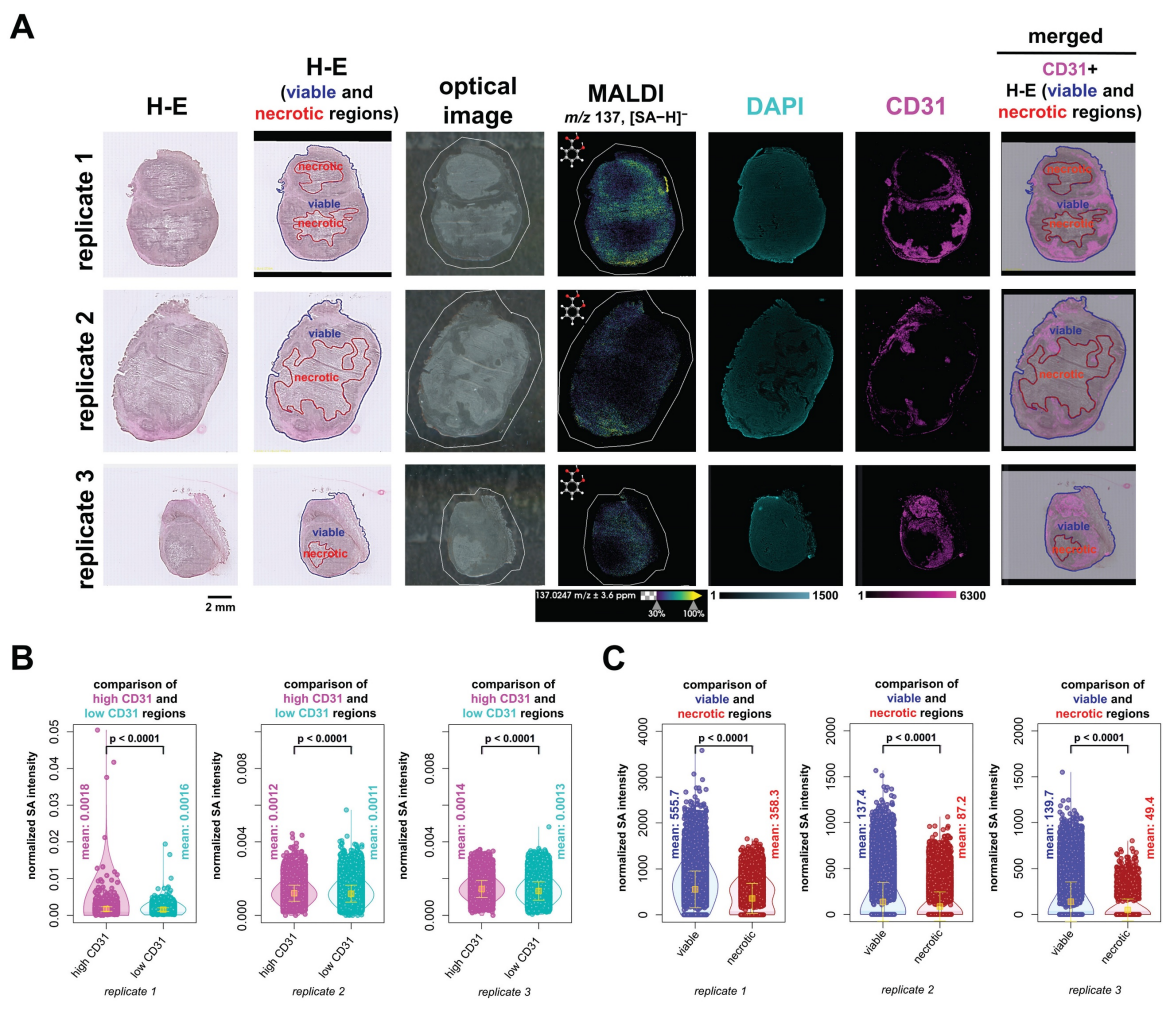
** QMALDI SA imaging data analysis integrated with H-E and CD31 immunofluorescence staining to quantify SA content in necrotic, viable, and vascularized tumor regions.** (**A**) The H-E and optical images are from the same tissue sections as the MALDI image, while the DAPI and CD31 images are from adjacent serial tissue sections. The figure includes three biological replicates of H-E, CD31, and DAPI-stained tumor sections, paired with MALDI images showing the spatial distribution of salicylic acid (SA) (*m/z* 137.02 Da, [M-H]⁻) in tissues from aspirin-treated mice. A merged image displaying the co-registration of the CD31 image with H-E-stained viable and necrotic regions in SUM159 tumors is shown in the leftmost images. (**B**) DAPI and CD31 images were co-registered with and down sampled to match the MALDI imaging data, enabling precise overlay and alignment. The integration of DAPI and CD31 immunofluorescence (IF) images with MALDI images was performed using the Weave software, allowing for a detailed spatial comparison across these different imaging modalities. The integrated image was analyzed by classifying regions into high-CD31(magenta) and low-CD31 (cyan) areas, followed by calculating the average normalized SA intensity for each region. (**C**) H-E-stained images of viable (blue) and necrotic (red) regions in SUM159 tumors were co-registered with MALDI images of SA using the SCiLS Lab software. The average normalized SA intensity was calculated for each region from these co-registered images. All presented measurements were performed by quantifying the SA intensity per pixel, where each open circle represents one pixel in the underlying violin plots. MALDI imaging was performed using a timsTOF fleX MALDI-2 instrument with norharmane matrix and peracetic acid additive at a 20 µm pixel size in negative ion mode. All results are also presented as mean ± standard deviation (SD) where the mean value for each group is indicated by yellow squares.

We were also interested in the biodistribution of SA with respect to tissue architecture and vasculature. To this extend, we performed H-E staining to evaluate anatomical tissue structures and CD31 IF staining to visualize vascular networks. We fused these imaging datasets, i.e., H-E, DAPI and CD31 IF, and MALDI imaging, as illustrated in Figure 5 and further analyzed the fused datasets. This was achieved by co-registering and scaling the MALDI images and CD31 and DAPI fluorescence images to the same pixel size which fused their spatial and intensity information. The higher resolution IF images were downscaled to the same resolution as the co-registered MALDI images, while ensuring that the corresponding pixels from different images were spatially matched. As a result, each pixel contained both the MALDI imaging information of SA abundance and the CD31 and DAPI fluorescence intensity (Figure 6A). We further analyzed and compared the SA intensity per pixel in areas classified as highly *versus* lowly vascularized regions based on their CD31 fluorescence intensity (Figure 6B). In all three biological replicates, SA levels were significantly higher in highly vascularized CD31-positive regions as compared to lowly vascularized CD31-negative regions. Additionally, H-E staining combined with MALDI imaging analysis consistently revealed higher SA levels in viable tumor regions compared to necrotic areas. This difference was also statistically significant across all three biological replicates (Figure 6C). Overall, our data demonstrates that SA predominantly accumulated in highly vascularized CD31-positive regions of viable tumor tissue.

**Figure 7:**
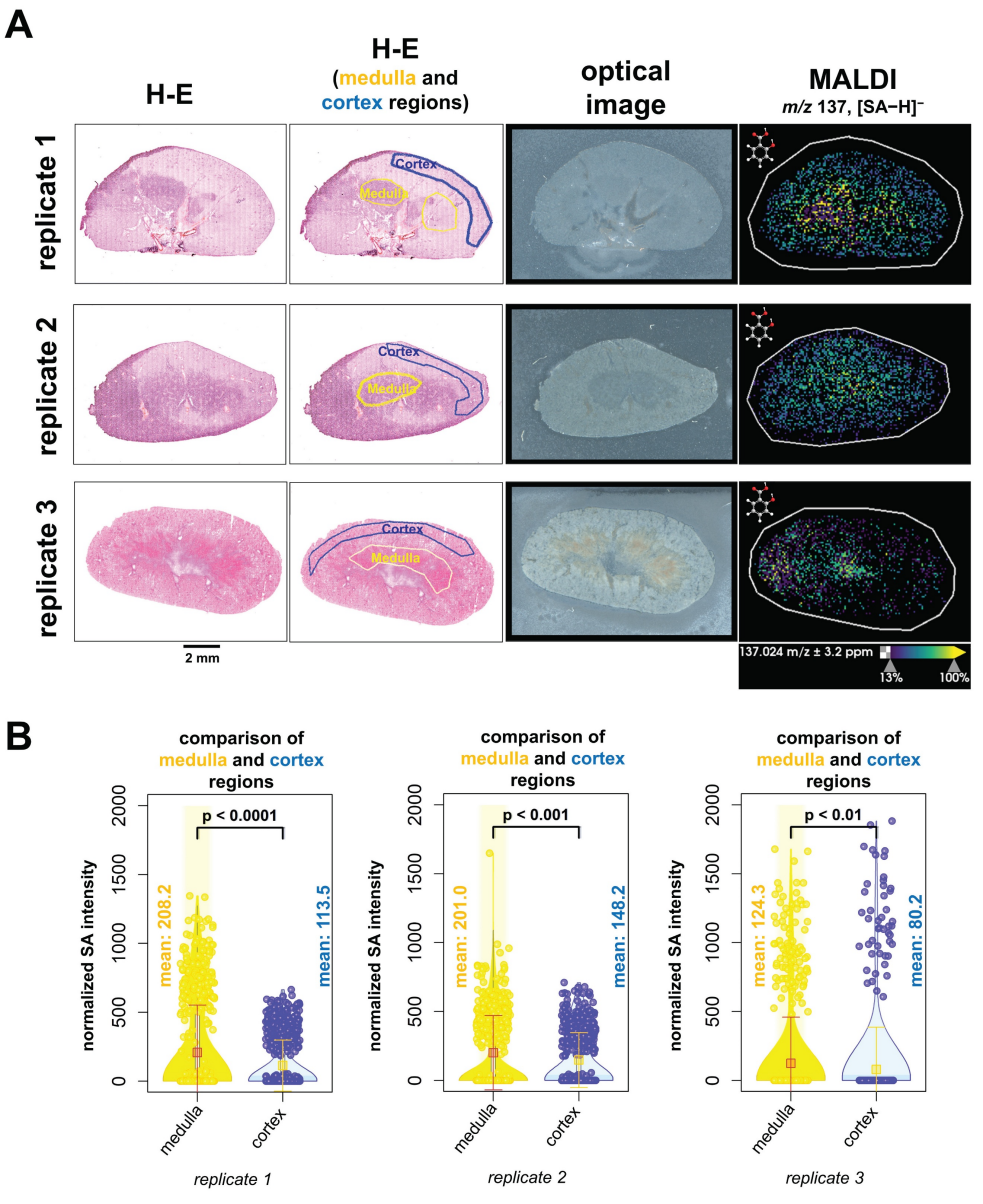
**QMALDI SA imaging data analysis integrated with H-E staining to quantify SA content in anatomical kidney regions.** (**A**) The H-E and optical images are from the same tissue sections as the MALDI image. The figure includes three biological replicates of H-E-stained kidney sections, paired with MALDI images showing the spatial distribution of salicylic acid (SA) (*m/z* 137.02 Da, [M-H]⁻) in tissues from aspirin-treated mice. (**B**) H-E-stained images of medulla (yellow) and cortex (blue) regions in kidneys were co-registered with MALDI images of SA using SCiLS Lab software. The average normalized SA intensity was calculated for each region from these co-registered images. All presented measurements were performed by quantifying the SA intensity per pixel, where each open circle represents one pixel in the underlying violin plots. MALDI imaging was performed using a timsTOF fleX MALDI-2 instrument with norharmane matrix and peracetic acid additive at a 20 µm pixel size in negative ion mode. All results are also presented as mean ± standard deviation (SD) where the mean value for each group is indicated by yellow squares.

We next evaluated the spatial distribution of SA abundance within kidney tissues and analyzed the fused data of MALDI imaging combined with H-E staining (Figure 7A). H-E-stained kidney tissue sections were annotated as medulla regions outlined with a yellow line and cortex regions outline with a blue line. Only regions with definitive anatomy were outlined. Our results revealed that higher levels of SA were detected in the medulla region than in the cortex region at the time of animal sacrifice, which was 60 minutes post-injection (Figure 7B). We therefore conclude that SA accumulated in the medulla region of kidneys at 60 minutes post-injection.

These QMALDI imaging findings are in good agreement with our previous CEST MRI study 9. The spatial distribution of high SA levels in the kidney medulla and in the SUM159 tumor rim, which was CD31-positive and viable, as determined by QMALDI imaging in this study (Figure 6 and 7) is consistent with the SA distribution patterns we previously observed using SA CEST MRI 9. Also, we observed a much higher signal contrast in kidney tissue than tumor tissue here, i.e., about 4 times higher, which is comparable to that observed by CEST MRI in our recent study. The QMALDI SA imaging approach developed in this study allows for a precise assessment of the spatial distribution of SA within organs and tumors, enabling researchers to evaluate their biodistribution at the microscopic level. The presented approach of analyzing QMALDI imaging data combined with fused H-E and IF data from the same or directly adjacent tissue sections, will significantly enhance biological studies of the biodistribution of contrast agents, chemotherapy agents, and anti-inflammatory drugs 4-9.

## Conclusions

We established a robust quantitative approach for targeted QMALDI imaging of SA by using nH matrix with 5 mM PAA as an additive and D_6_-SA as a deuterated internal standard. While previous studies have explored various approaches for quantitative MALDI imaging, including isotopically labeled internal standards 39 and fluorescence-assisted matrix application 20, the current work introduces several novel elements that significantly advance the field. First, we demonstrate that PAA at 5 mM concentration serves as a unique dual-function additive that both enhances signal intensity and reduces matrix noise through PAA-generated radicals from UV light exposure by the MALDI imaging laser. The PAA-mediated hydrogen bonding with nH matrix generates exceptionally uniform, small crystal structures (0.64 ± 0.05 μm²), significantly enhancing both ionization efficiency and analytical reproducibility. This optimized microcrystalline morphology is critical for achieving consistent analyte desorption/ionization across tissue sections. This PAA-assisted MALDI imaging technique allows for precise quantification of SA, an aspirin metabolite and active CEST MRI contrast agent, while accurately mapping its spatial distribution in heterogeneous biological tissues.

This new QMALDI imaging technique was applied to quantitatively measuring SA in liver, kidney, and SUM159 TNBC tumor tissues in mice at 60 minutes following intravenous injection of aspirin. QMALDI imaging was able to quantify SA tissue concentrations of 133.0 ± 44.6 pmol/mm² in the liver, 140.3 ± 23.3 pmol/mm² in the kidney, and 34.5 ± 10.8 pmol/mm² in the SUM159 TNBC tumor from three biological replicates each. High consistency observed between technical and biological replicates in this study reinforces the reliability of the newly developed QMALDI imaging technique for detecting the spatial distribution and absolute quantitation of SA in tissue sections.

Furthermore, we observed high SA in the kidney medulla and the CD31-positive, highly vascularized and viable regions in the tumor rim. This was possible by co-registration, scaling, and pixel matching of the MALDI imaging data with H-E stained, and CD31 and DAPI IF stained slide-scanned images of the same and adjacent tissue sections. These findings match our earlier findings with SA CEST MRI 9. Targeted QMALDI SA imaging, as well as other similar QMALDI imaging approaches, allows us to accurately assess the spatial distribution and absolute concentration of drugs, drug metabolites, and contrast agents in various tissues for advancing our understanding of their biodistribution and biomedical effects, as well as helping to eventually move these approaches into clinical applications.

## Supplementary Material

Supplementary figures.

## Figures and Tables

**Table 1:** Impact of PAA concentration on average spectra of dried SA droplets

Mode	Analyte	Tissue Type	0 mM PAA (SA intensity)^a^	1 mM PAA (SA intensity)^a^	5 mM PAA (SA intensity)^a^	10 mM PAA (SA intensity)^a^	20 mM PAA (SA intensity)^a^
Negative	SA	Liver	48.9 ± 18.6	66.5 ± 42.3	157.3 ± 26.2	37.7 ± 15.1	21.7 ± 24.5
		Kidney	379.4 ± 125.3	335.3 ± 57.7	692.3 ± 165.2	121.1 ± 21.3	42.1 ± 22.3
		SUM159	333.7 ± 42.5	424.0 ± 78.1	550.4 ± 24.1	164.8 ± 17.3	267.7 ± 23.1

^a^: n = 3

**Table 2:** QMALDI imaging analysis of SA in various tissues

Mode	Analyte	Tissue Type	LOD (pmol/mm^2^)	LOQ (pmol/mm^2^)	Technical Repeats (pmol/mm^2^)^a^	Biological Repeats (pmol/mm^2^)^a^
Negative	SA	Liver	0.3	0.9	141.9 ± 22.6	133.0 ± 44.6
		Kidney	0.2	0.6	129.5 ± 7.8	140.3 ± 23.3
		SUM159	0.4	1.3	50.4 ± 3.0	34.5 ± 10.8

^a^: n = 3

## Abbreviations

COX: cyclooxygenase
ASA: aspirin
SA: salicylic acid
CEST: chemical exchange saturation transfer
MRI: magnetic resonance imaging
DTPA: diethylenetriamine pentaacetate
DAPI: 4′,6-diamidino-2-phenylindole
MALDI-MS: matrix-assisted laser desorption/ionization mass spectrometry
QMALDI: quantitative MALDI
TNBC: triple-negative breast cancer
H-E: hematoxylin and eosin
PAA: peracetic acid
nH: norharmane
SAG: salicyl acyl glucuronide
SPG: salicyl phenolic glucuronide
SU: salicyluric acid
2,5-DHB: 2,5-dihydroxybenzoic acid
PFA: paraformaldehyde
PBS: phosphate-buffered saline
BSA: bovine serum albumin
TFA: trifluoroacetic acid
ITO: indium tin oxide
SEM: scanning electron microscopy
MSI: MALDI-MS imaging
IF: immunofluorescence
D_6_-SA: D_6_-salicylic acid
FL: fluorescence
SD: standard deviation
