# Supplementary material for: Quantitative MALDI imaging of aspirin metabolites in mouse models of triple-negative breast cancer
Source: Theranostics. 2025 Aug 22;15(18):9359–74. doi: 10.7150/thno.116819 (PMC12486018; doi:10.7150/thno.116819)
Supplement: Supplementary file 1 — Supplementary figures. [file thnov15p9359s1.pdf]

Supporting Information

Quantitative MALDI imaging of aspirin metabolites in mouse models of triple-negative breast cancer

Tae-Hun Hahm,[a,b] Dalton R. Brown,[a] Caitlin M. Tressler,[a,b] Thao Tran,[c] Alice Ly,[c] Arvind P. Pathak[b], Michael T. McMahon,[b,e] Kristine Glunde,*,[a,b,d,f]

[a] The Johns Hopkins Applied Imaging Mass Spectrometry Core and Service Center, Division of Cancer Imaging Research,

[b] The Russell H. Morgan Department of Radiology and Radiological Science, The Johns Hopkins University School of Medicine, Baltimore, MD 21205, USA

[c] Aspect Analytics NV, C-mine 12, 3600 Genk, Belgium

[d] The Sidney Kimmel Comprehensive Cancer Center, The Johns Hopkins University School of Medicine, Baltimore, MD 21205, USA Aspect

[e] F.M. Kirby Research Center for Functional Brain Imaging, Kennedy Krieger Institute, Baltimore, MD 21205, USA

[f] Department of Biological Chemistry, The Johns Hopkins University School of Medicine, Baltimore, MD 21205, USA

Table of Contents

Supplementary Figures

Figure S1. Effect of PAA concentration on SA fragment ion (*m/z* 93.03 Da) detection in tissue samples............................... S3

Figure S2. Comparison of experimental conditions: Matrix and SA signals in liver tissues with or without PAA S4

Figure S3. Comparison of experimental conditions: Matrix and SA signals in kidney tissues with or without PAA S5

Figure S4. Comparison of experimental conditions: Matrix and SA signals in SUM159 tumor tissues with or without PAA S6

Figure S5. MS/MS spectrum of *m/z* 154.97 in negative ion mode, generated from the norharmane matrix S7

Figure S6. MS/MS spectrum of *m/z* 248.96 in negative ion mode, generated from the norharmane matrix S8

Figure S7. Hydroxyl radical scavenging experiment to validate PAA mechanism in MALDI imaging S9-S10

Figure S8. MALDI imaging of aspirin distribution in tissue sections from aspirin treated versus untreated control mice... S11

Figure S9. MALDI imaging of 2,5-DHB distribution in tissue sections from aspirin treated versus untreated control mice S12

Figure S10. MALDI imaging of SAG distribution in tissue sections from aspirin treated versus untreated control mice..... S13

Figure S11. MALDI imaging of SPG distribution in tissue sections from aspirin treated versus untreated control mice.... S14

Figure S12. MALDI imaging of SU distribution in tissue sections from aspirin treated versus untreated control mice...... S15

Figure S13. MS/MS spectra in negative ion mode for *m/z* 137.02, identified as SA S16

Figure S14. MS/MS spectra in negative ion mode for *m/z* 179.03, identified as aspirin S17

Figure S15. MS/MS spectra in negative ion mode for *m/z* 153.02, identified as 2,5-DHB S18

Figure S16. Comprehensive workflow map for SA quantification in tissue samples by MALDI-MSI S19

Figure S17. MALDI imaging analysis of sprayed on D_6-SA and tissue-contained SA in liver, kidney, and SUM159 tumor tissue sections... S20

Figure S18. Microscopic images of liver sections stained with Hematoxylin-Eosin (H-E), alongside MALDI imaging..... S21

Supplementary Figures

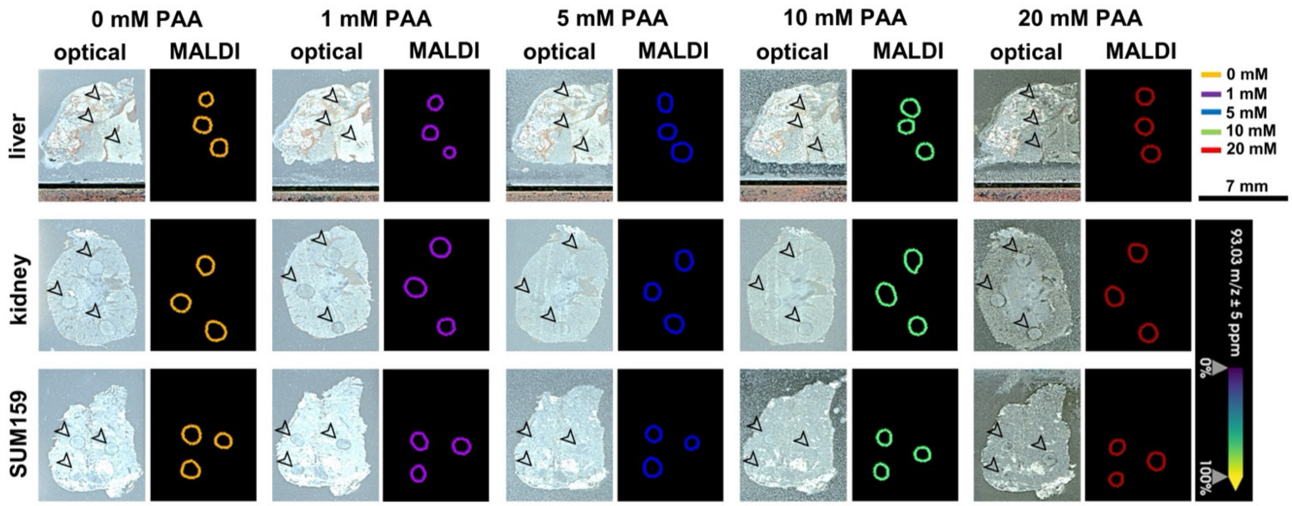

Figure S1. **Effect of PAA concentration on SA fragment ion (*m/z* 93.03 Da) detection in tissue samples.** Optical and MALDI images of tissue sections spotted with dried droplets of 1 mM salicylic acid (SA) followed by application of norharmane (nH), combined with varying concentrations of peracetic acid (PAA) (0 mM, 1 mM, 5 mM, 10 mM, and 20 mM). Black arrows indicate the locations of the dried SA droplets in the optical images. Matrix-Assisted Laser Desorption/Ionization (MALDI) images were acquired at 100 µm pixel size in negative ion mode using a timsTOF fleX MALDI-2 instrument. The data demonstrate that the SA fragment ion at $[M{-}H]^-$ = *m/z* 93.03 Da was not detected in the dried droplets spotted on tissue sections.

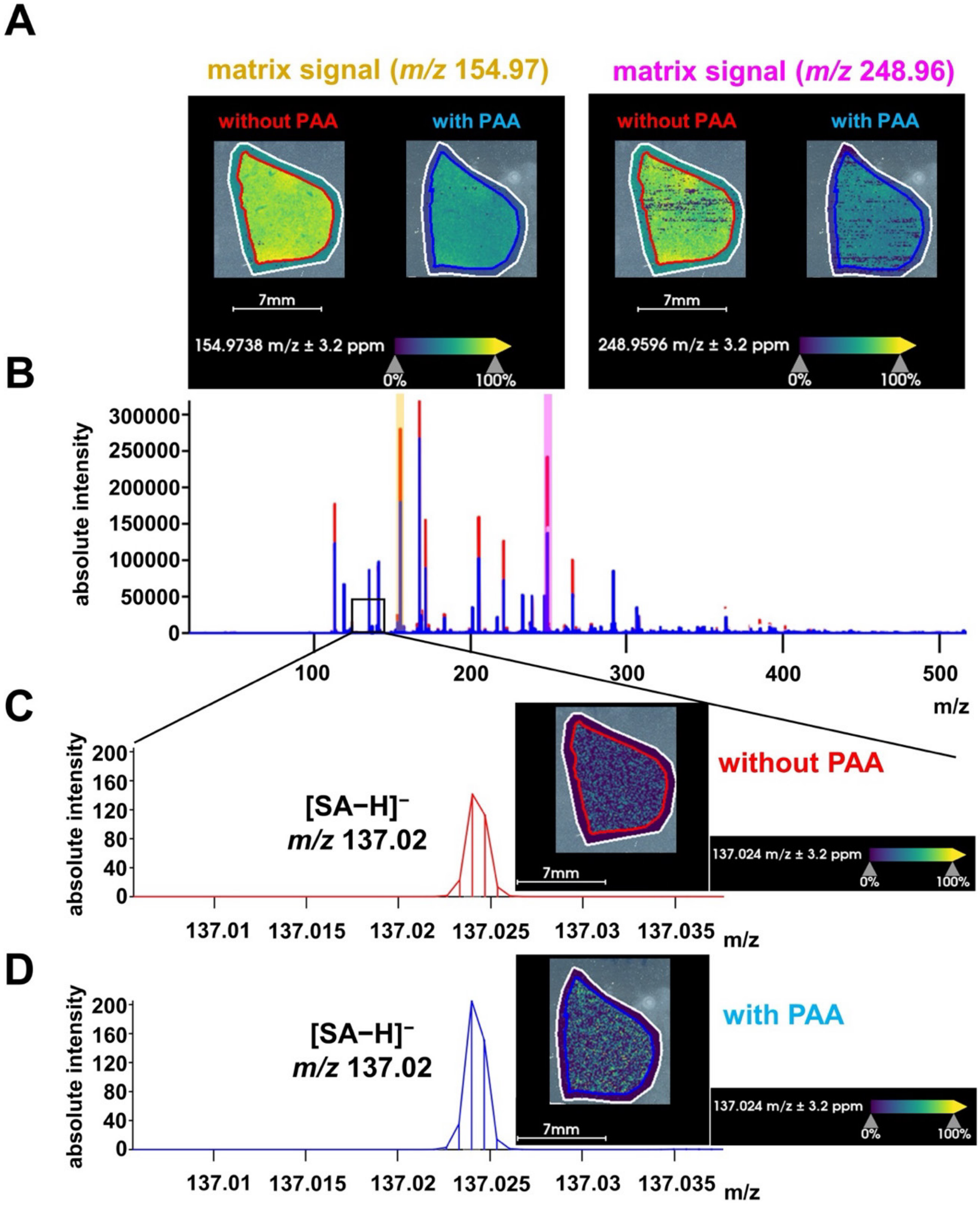

Figure S2. Comparison of experimental conditions: Matrix and SA signals in liver tissues with or without PAA. (**A**) MALDI images showing the two highest matrix background signals at *m/z* 154.97 Da and *m/z* 248.96 Da, and (**B**) corresponding average spectra ranging from *m/z* 0–500 Da in liver sections from aspirin-treated mouse mice following norharmane application, either with (blue spectra) or without (red spectra) 5 mM PAA. The MALDI images were acquired at 100 µm pixel size in negative ion mode using a timsTOF fleX MALDI-2 instrument. The data demonstrate that the $[M-H]^-$ ion of SA at *m/z* 137.02 Da was detected in tissue sections from aspirin treated mice after norharmane application (**C**) with or (**D**) without 5 mM PAA, which resulted in a higher SA signal intensity when PPA was applied.

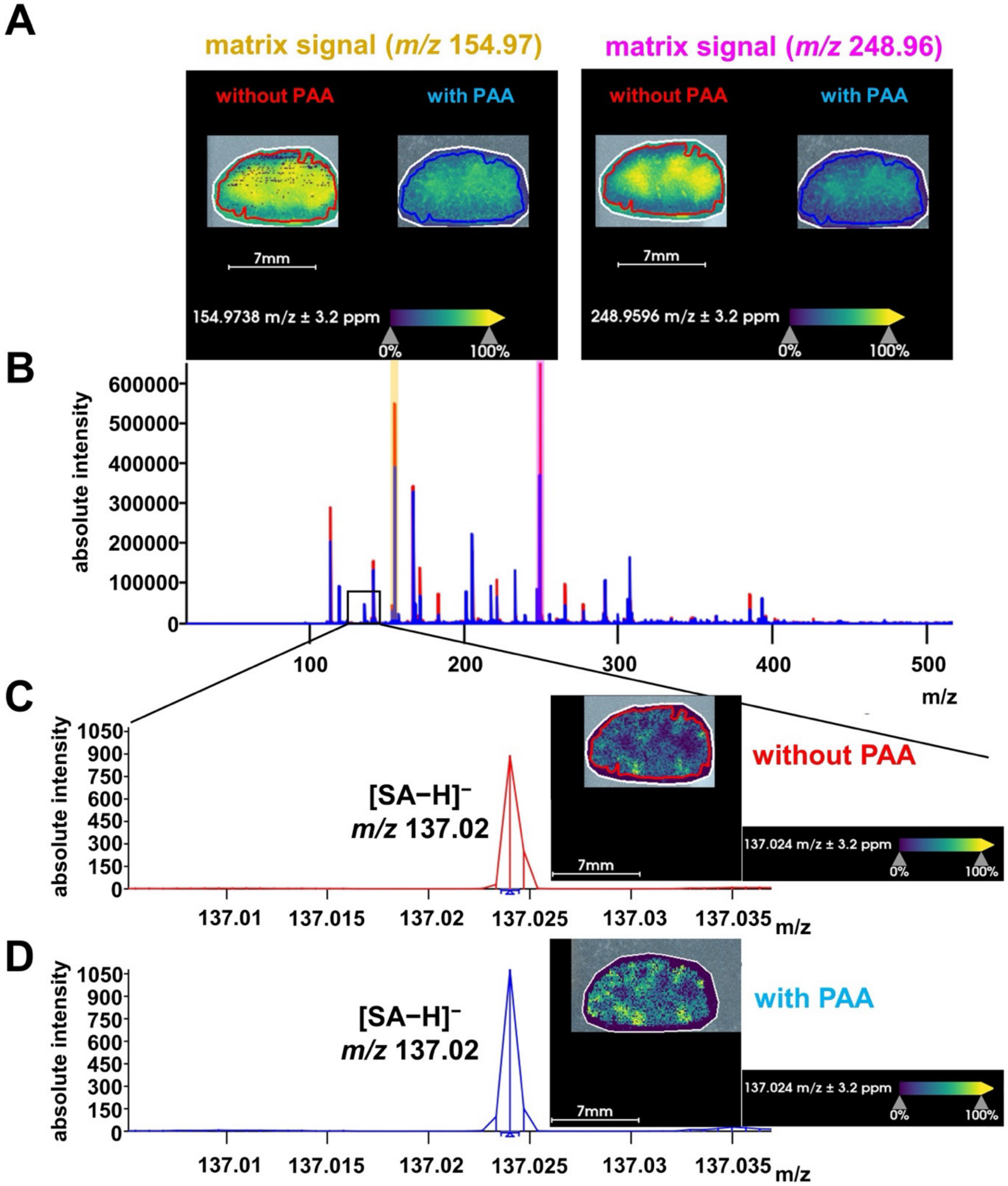

<u>Figure S3.</u> Comparison of experimental conditions: Matrix and SA signals in kidney tissues with or without PAA. (**A**) MALDI images showing the two highest matrix background signals at *m/z* 154.97 Da and *m/z* 248.96 Da, and (**B**) corresponding average spectra ranging from *m/z* 0–500 Da in kidney sections from aspirin-treated mice following norharmane application, either with (blue spectra) or without (red spectra) 5 mM PAA. The MALDI images were acquired at 100 µm pixel size in negative ion mode using a timsTOF fleX MALDI instrument. The data demonstrate that the $[M-H]^-$ ion of SA at *m/z* 137.02 Da was detected in tissue sections from aspirin treated mice after norharmane application (**C**) with or (**D**) without 5 mM PAA, which resulted in a higher SA signal intensity when PAA was applied.

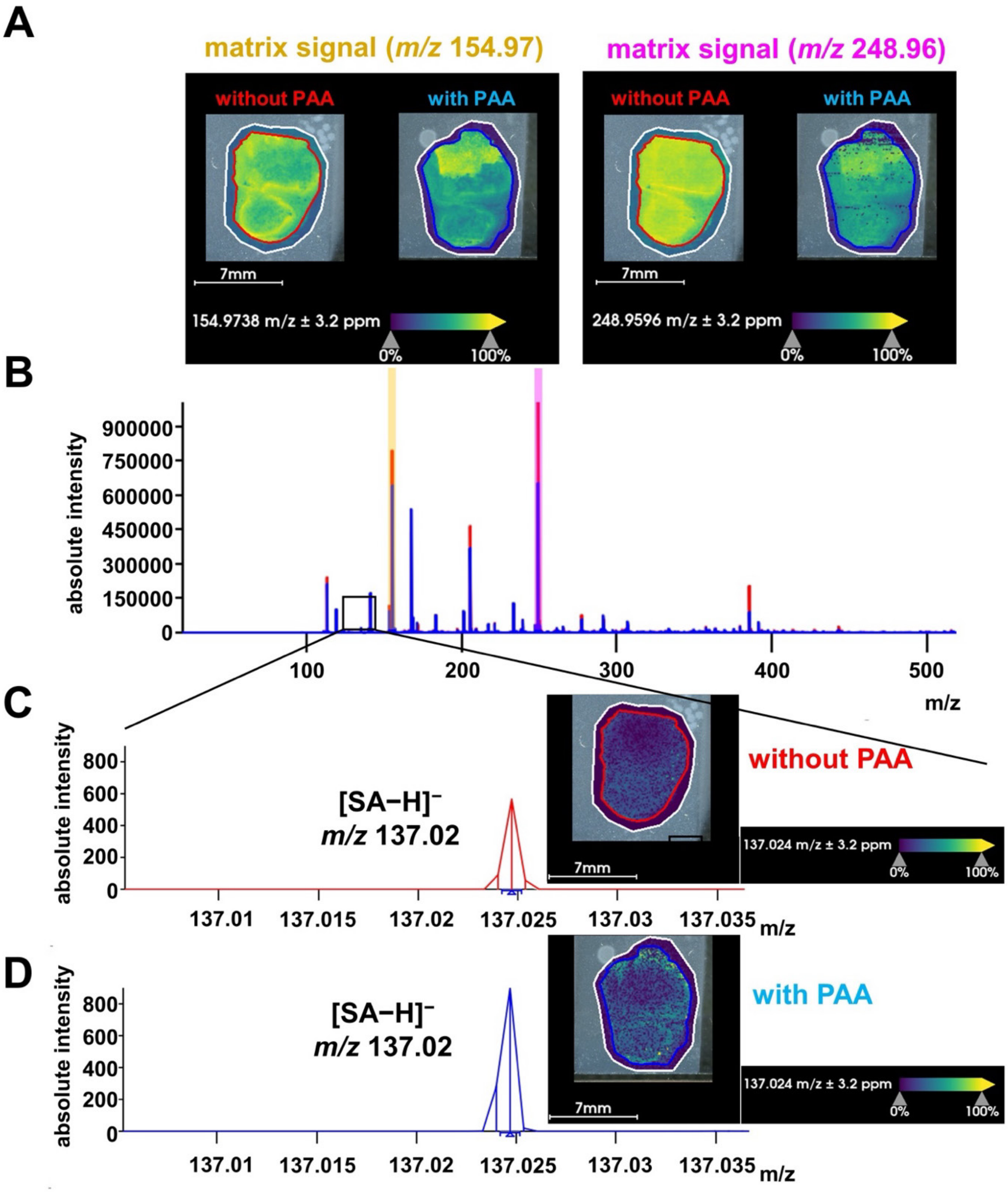

<u>Figure S4.</u> Comparison of experimental conditions: Matrix and SA signals in SUM159 tumor tissues with or without PAA. (**A**) MALDI images showing the two highest matrix background signals at *m/z* 154.97 Da and *m/z* 248.96 Da, and (**B**) corresponding average spectra ranging from *m/z* 0–500 Da in SUM159 tumor sections from aspirin-treated mice tissue following norharmane application, either with (blue spectra) or without (red spectra) 5 mM PAA. The MALDI images were acquired with a 100 µm pixel size in negative ion mode using a timsTOF fleX MALDI-2 instrument. The data demonstrate that the $[M{-}H]^-$ ion of SA at *m/z* 137.02 Da was detected in tissue sections from aspirin treated mice after norharmane application (**C**) with or (**D**) without 5 mM PAA, which resulted in a higher SA signal intensity when PPA was applied.

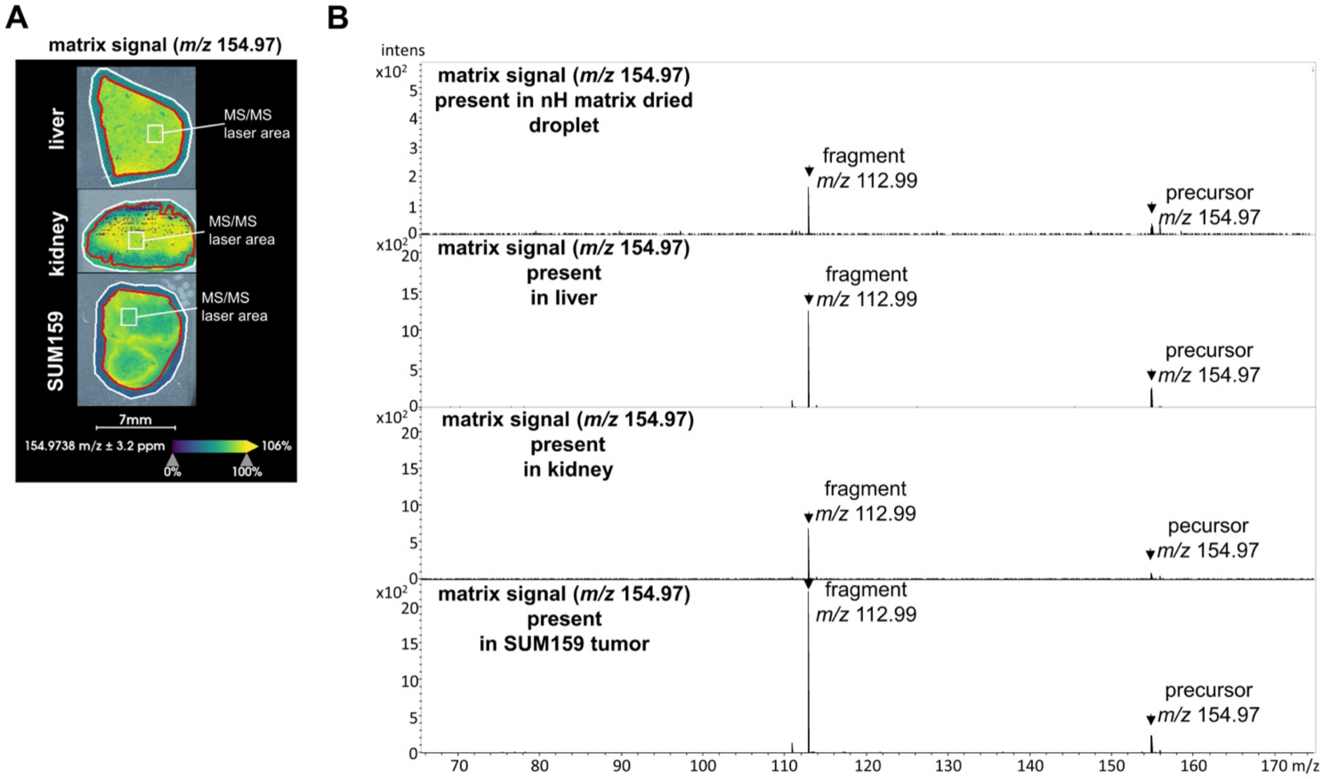

<u>**Figure S5.**</u> **MS/MS spectrum of *m/z* 154.97 in negative ion mode, generated from the norharmane matrix.** (**A**) The MS/MS laser area used for the analysis is highlighted with a white box. The (**A**) spatial distribution and (**B**) precursor peak at *m/z* 154.97 Da, and its fragment ion at *m/z* 112.99 Da are shown for a dried norharmane (nH) droplet, as well as for liver, kidney, and SUM159 tumor sections.

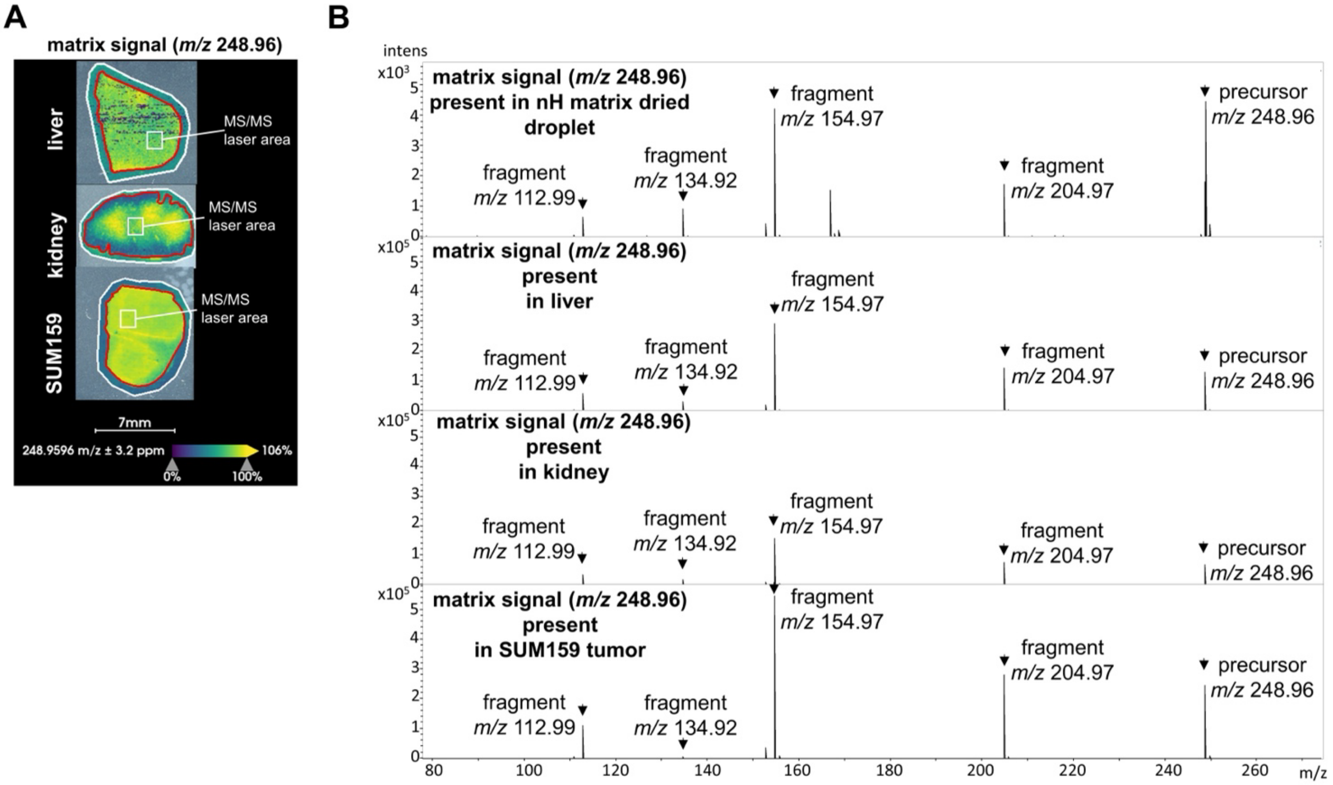

<u>**Figure S6.**</u> **MS/MS spectrum of *m/z* 248.96 in negative ion mode, generated from the norharmane matrix.** (**A**) The MS/MS laser area used for the analysis is highlighted with a white box. The (**A**) spatial distribution and (**B**) precursor peak at *m/z* 248.96 Da, and its fragment ions at *m/z* 112.99, 134.92, 154.97, and 204.97 Da are shown for a dried norharmane (nH) droplet, as well as for liver, kidney, and SUM159 tumor sections.

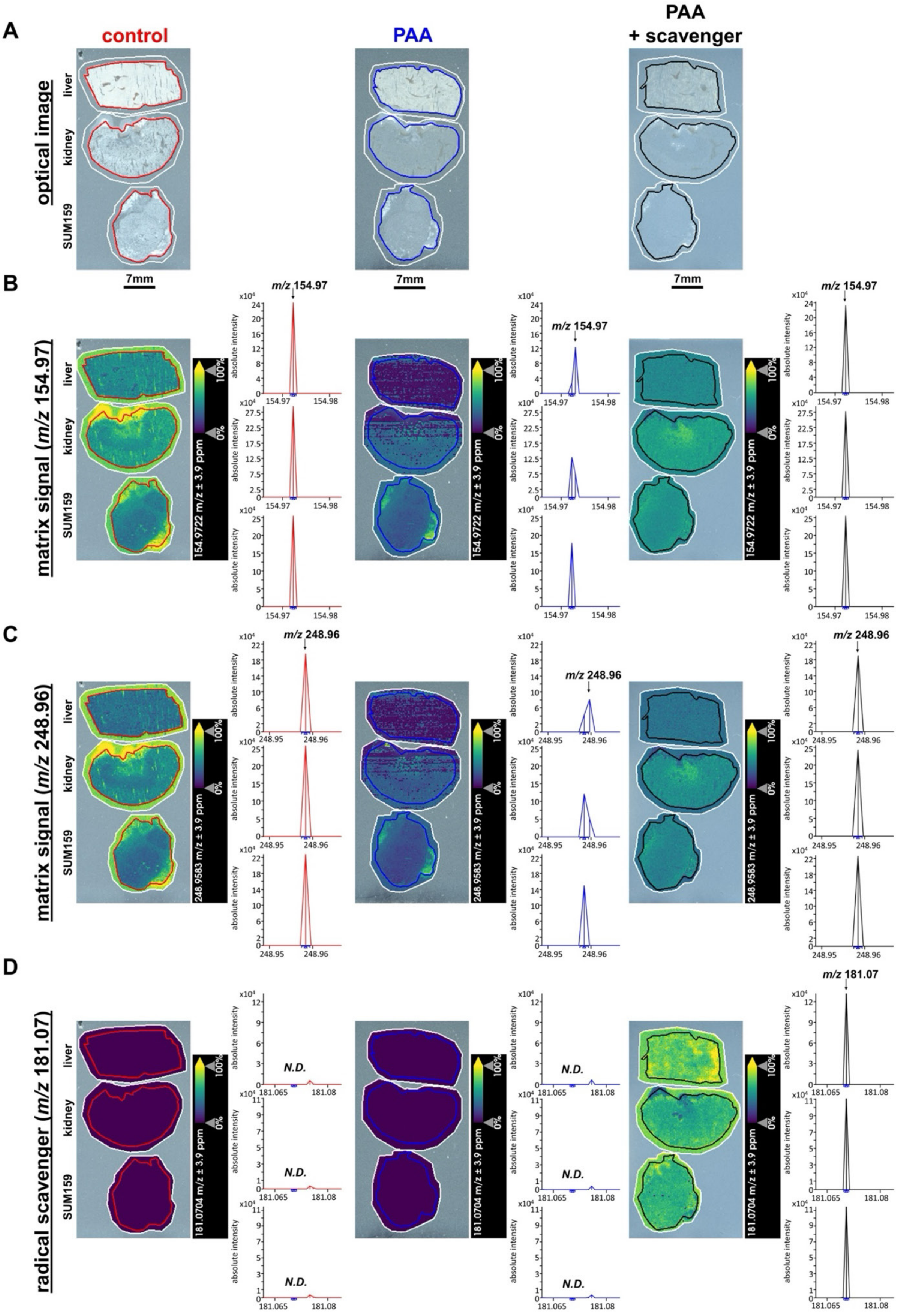

A
control
PAA
PAA + scavenger
optical image
liver
kidney
SUM159
7mm
B
matrix signal (m/z 154.97)
m/z 154.97
154.9722 m/z ± 3.9 ppm
absolute intensity
C
matrix signal (m/z 248.96)
m/z 248.96
248.9583 m/z ± 3.9 ppm
D
radical scavenger (m/z 181.07)
m/z 181.07
181.0704 m/z ± 3.9 ppm
N.D.
100%
0%

<u>Figure S7.</u> Hydroxyl radical scavenging experiment to validate PAA mechanism in MALDI imaging. (**A**) Optical images of tissue sections after matrix application showing crystal morphology under three experimental conditions: Control (nH matrix only), PAA (nH matrix + 5 mM PAA), and PAA + scavenger (nH matrix + 5 mM PAA + 100 mM D-mannitol). Images demonstrate uniform matrix deposition across all conditions in liver, kidney, and SUM159 tumor tissues. (**B**) MALDI imaging and corresponding mass spectra of matrix background signal at *m/z* 154.97 across the three experimental conditions. Left panels show spatial maps of the two indicated matrix and scavenger *m/z* values, while right panels display representative spectra from liver, kidney, and SUM159 tumor tissues. Adding PAA to the matrix results in reduced matrix signal intensity compared to matrix-only control, while with the addition of the radical scavenger D-mannitol and PAA to the matrix, matrix signals are restored, indicating hydroxyl radical involvement in matrix ion suppression. (**C**) MALDI imaging and corresponding mass spectra of matrix background signal at *m/z* 248.96 across the three experimental conditions. Similar to panel B, the data show PAA-induced signal reduction and D-mannitol-mediated signal recovery, providing additional evidence for hydroxyl radical-mediated matrix background suppression. (**D**) MALDI imaging and corresponding mass spectra of D-mannitol ($[M-H]^-$, *m/z* 181.07) confirms that the presence of D-mannitol for PAA + scavenger. Ion intensity maps and spectra from MALDI imaging data of liver, kidney, and SUM159 tumor tissues confirm the successful incorporation and detection of the hydroxyl radical scavenger D-mannitol in tissue sections to which it was applied in the experimental group of PAA + scavenger. **N.D.*: Not detected.

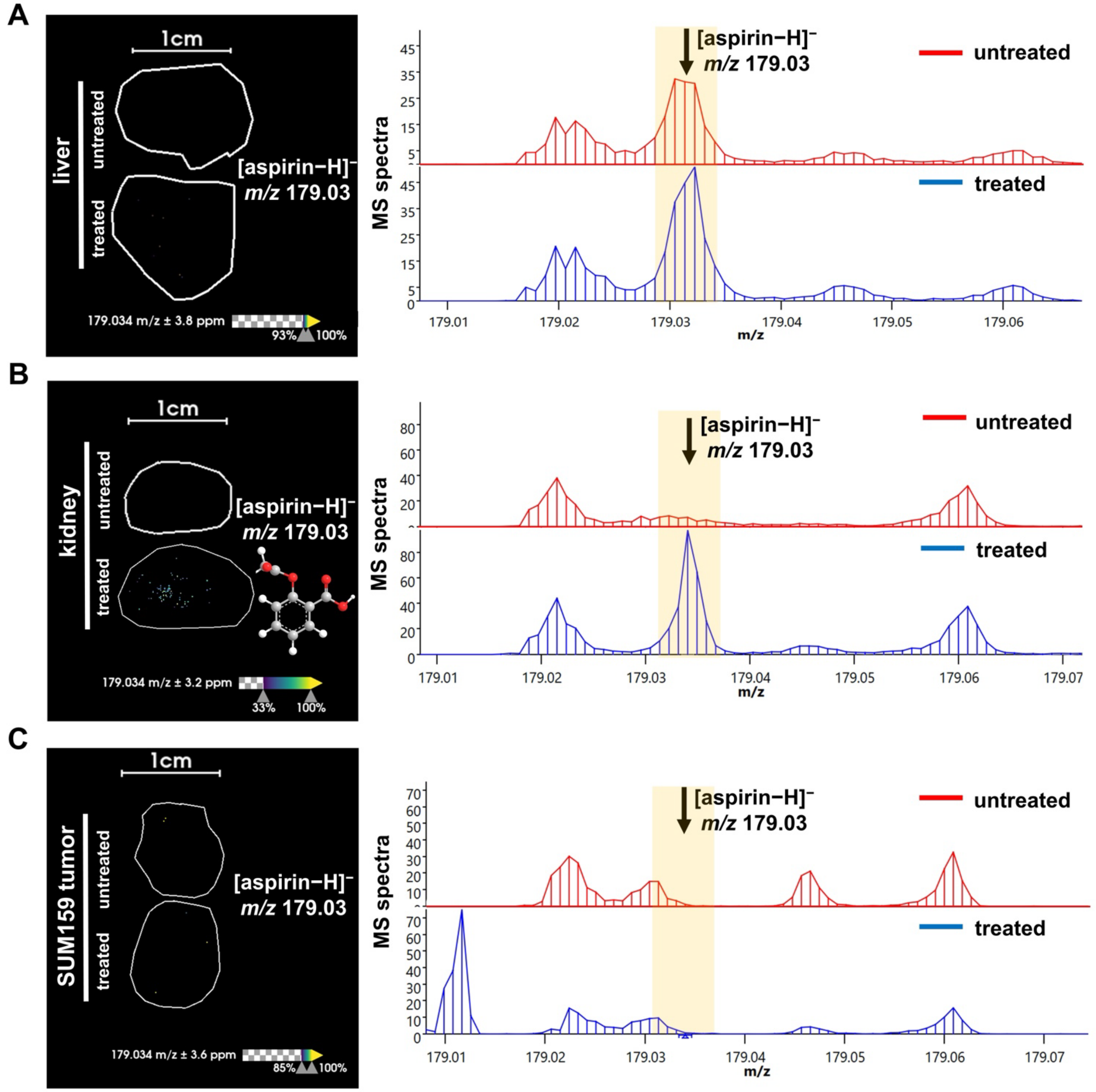

<u>Figure S8.</u> MALDI imaging of aspirin distribution in tissue sections from aspirin treated versus untreated control mice. MALDI imaging and corresponding average spectra of aspirin distribution in representative (**A**) liver, (**B**) kidney, and (**C**) SUM159 tumor sections from 300 mM aspirin-treated (bottom, blue spectra) and untreated control (top, red spectra) mice. The data clearly show that the $[M{-}H]^-$ ion of aspirin at *m/z* 179.03 Da was detected in kidney tissue sections, but not in tumor and liver sections from aspirin treated mice, while tissues from untreated mice showed no aspirin signal or comparable noise signal at this *m/z* value, indicating that the detected signal represents noise signal rather than specific aspirin.detection. The MALDI images were acquired at 100 µm pixel size in negative ion mode using a timsTOF fleX MALDI-2 instrument with norharmane matrix and PAA as an additive.

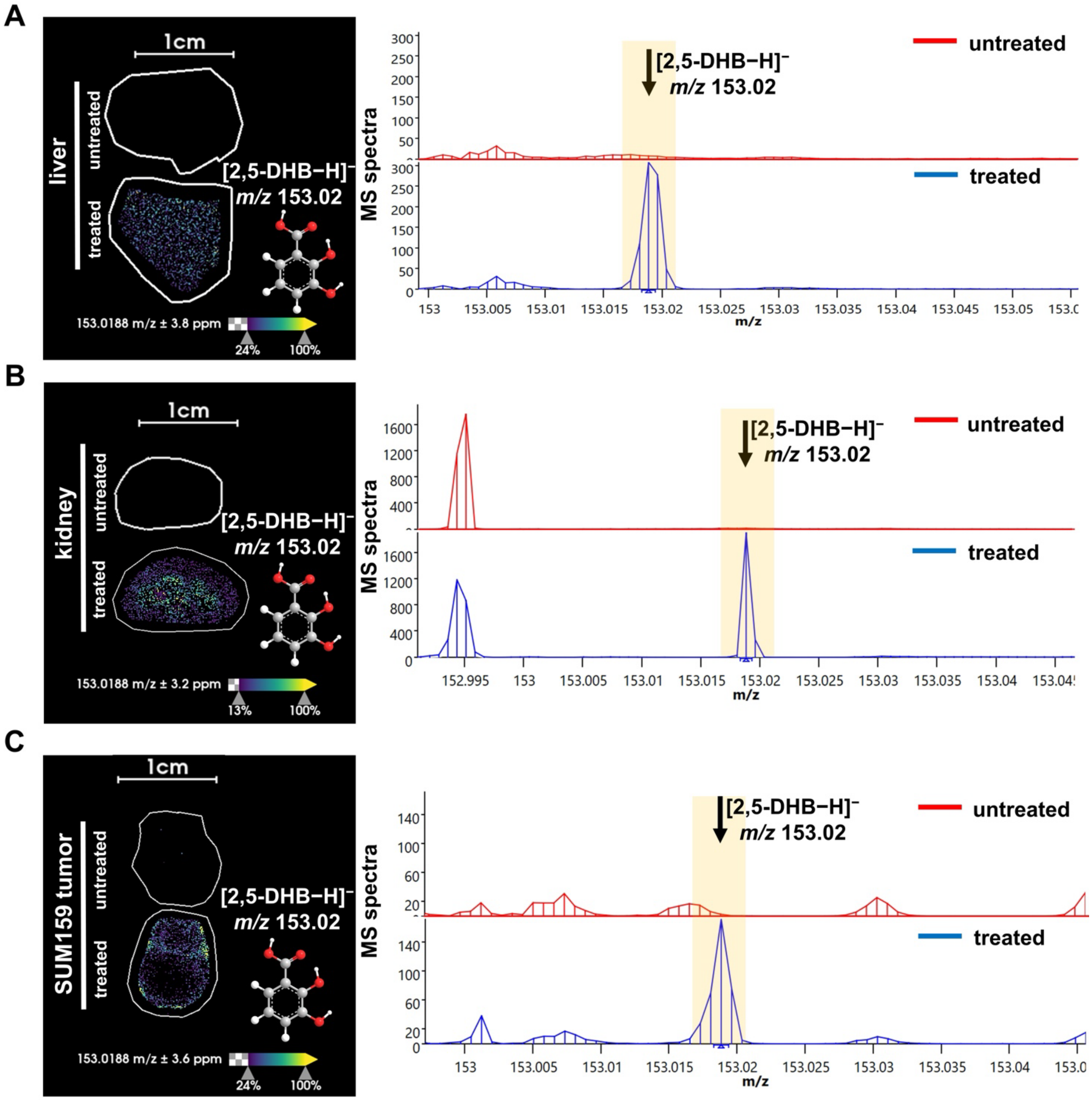

<u>Figure S9.</u> MALDI imaging of 2,5-DHB distribution in tissue sections from aspirin treated versus untreated control mice. MALDI imaging and corresponding average spectra of 2,5-DHB distribution in representative (**A**) liver, (**B**) kidney, and (**C**) SUM159 tumor sections from 300 mM aspirin-treated (bottom, blue spectra) and untreated control (top, red spectra) mice. The data clearly show that the $[M{-}H]^-$ ion of 2,5-DHB at *m/z* 153.02 Da was detected in SUM159 tumor, kidney, and liver tissue sections from aspirin treated mice, while it was absent in all tissues from untreated control mice. The MALDI images were acquired at 100 µm pixel size in negative ion mode using a timsTOF fleX MALDI-2 instrument with norharmane matrix and PAA as an additive.

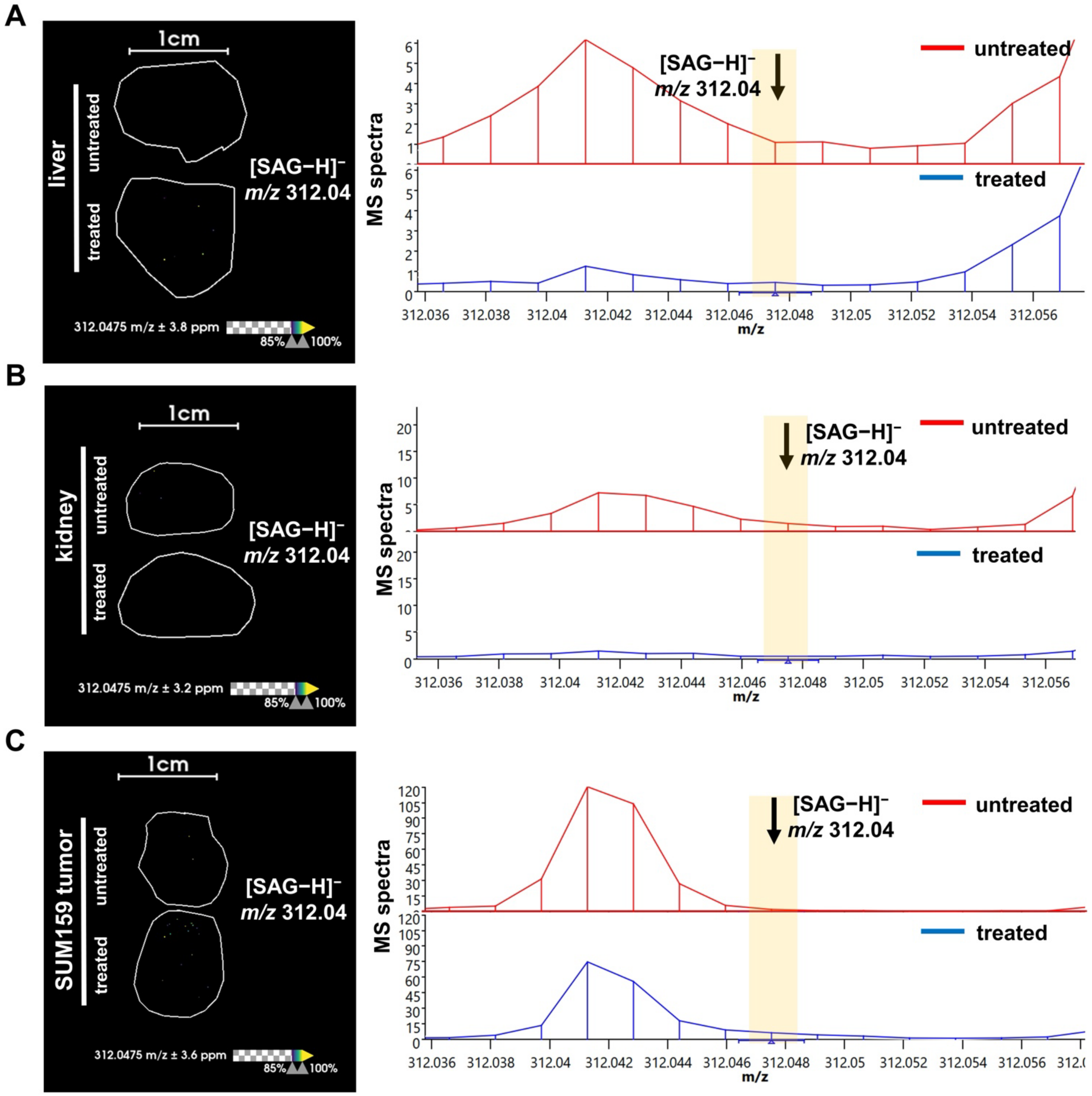

<u>Figure S10.</u> MALDI imaging of salicyl acyl-*β*-D-glucuronide (SAG) distribution in tissue sections from aspirin treated versus untreated control mice. MALDI imaging and corresponding average spectra of SAG distribution in (**A**) liver, (**B**) kidney, and (**C**) SUM159 tumor sections from 300 mM aspirin-treated (bottom, blue spectra) and untreated control (top, red spectra) mice. The data show that the $[M-H]^-$ ion of SAG at *m/z* 312.04 Da was not detected in any tissues, neither in tissues from aspirin treated mice nor in those from untreated control mice. The MALDI images were acquired at 100 µm pixel size in negative ion mode using a timsTOF fleX MALDI-2 instrument with norharmane matrix and PAA as an additive.

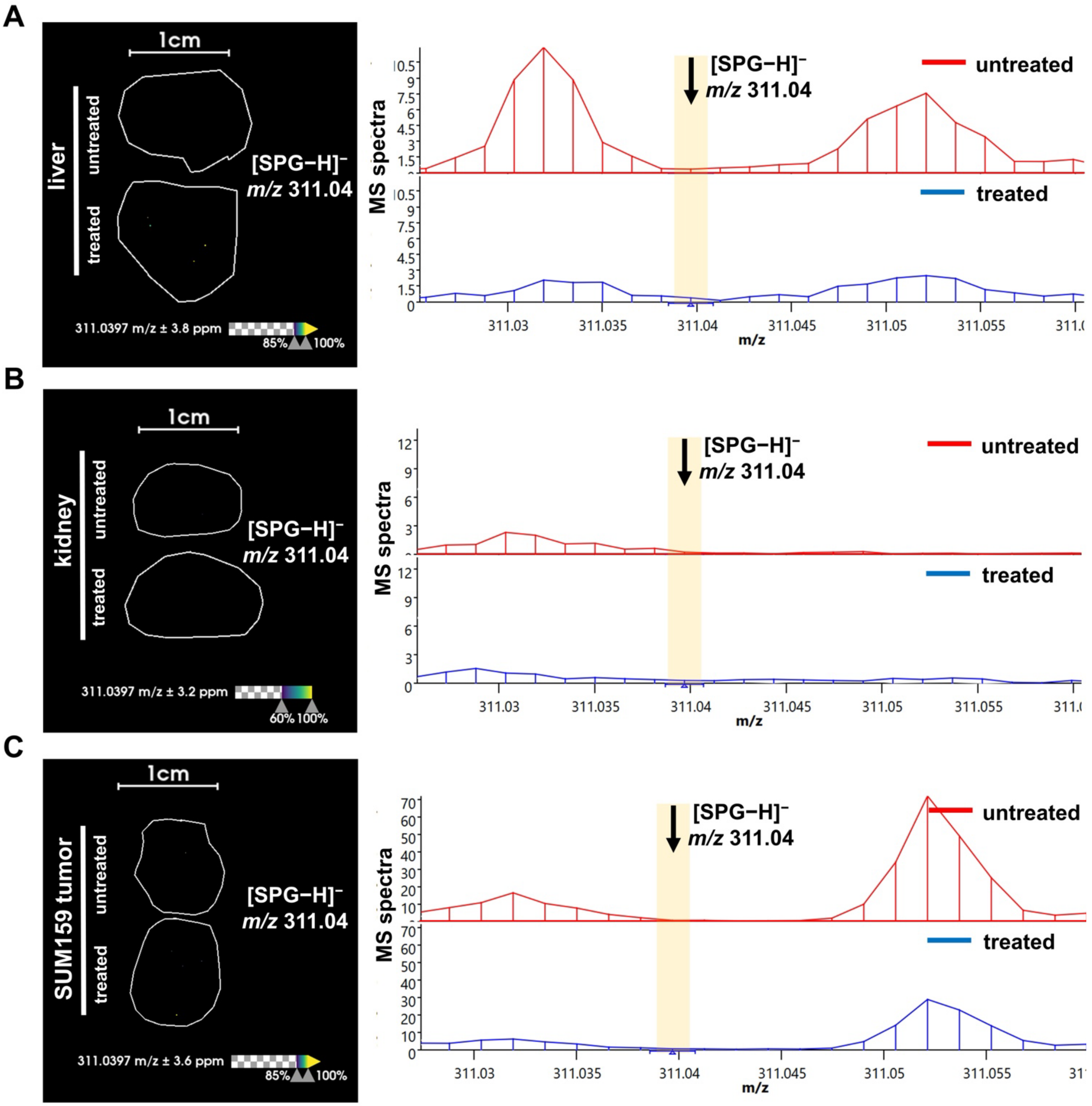

<u>Figure S11.</u> MALDI imaging of salicyl phenolic glucuronide (SPG) distribution in tissue sections from aspirin treated versus untreated control mice. MALDI imaging and corresponding average spectra of SPG distribution in representative (**A**) liver, (**B**) kidney, and (**C**) SUM159 tumor sections from 300 mM aspirin-treated (bottom, blue spectra) and untreated control (top, red spectra) tissues. The data show that the [M−H]⁻ ion of SPG at *m/z* 311.04 Da was not detected in any tissues, neither in tissues from aspirin treated mice nor in those from untreated control mice. The MALDI images were acquired at 100 µm pixel size in negative ion mode using a timsTOF fleX MALDI-2 instrument with norharmane matrix and PAA as an additive.

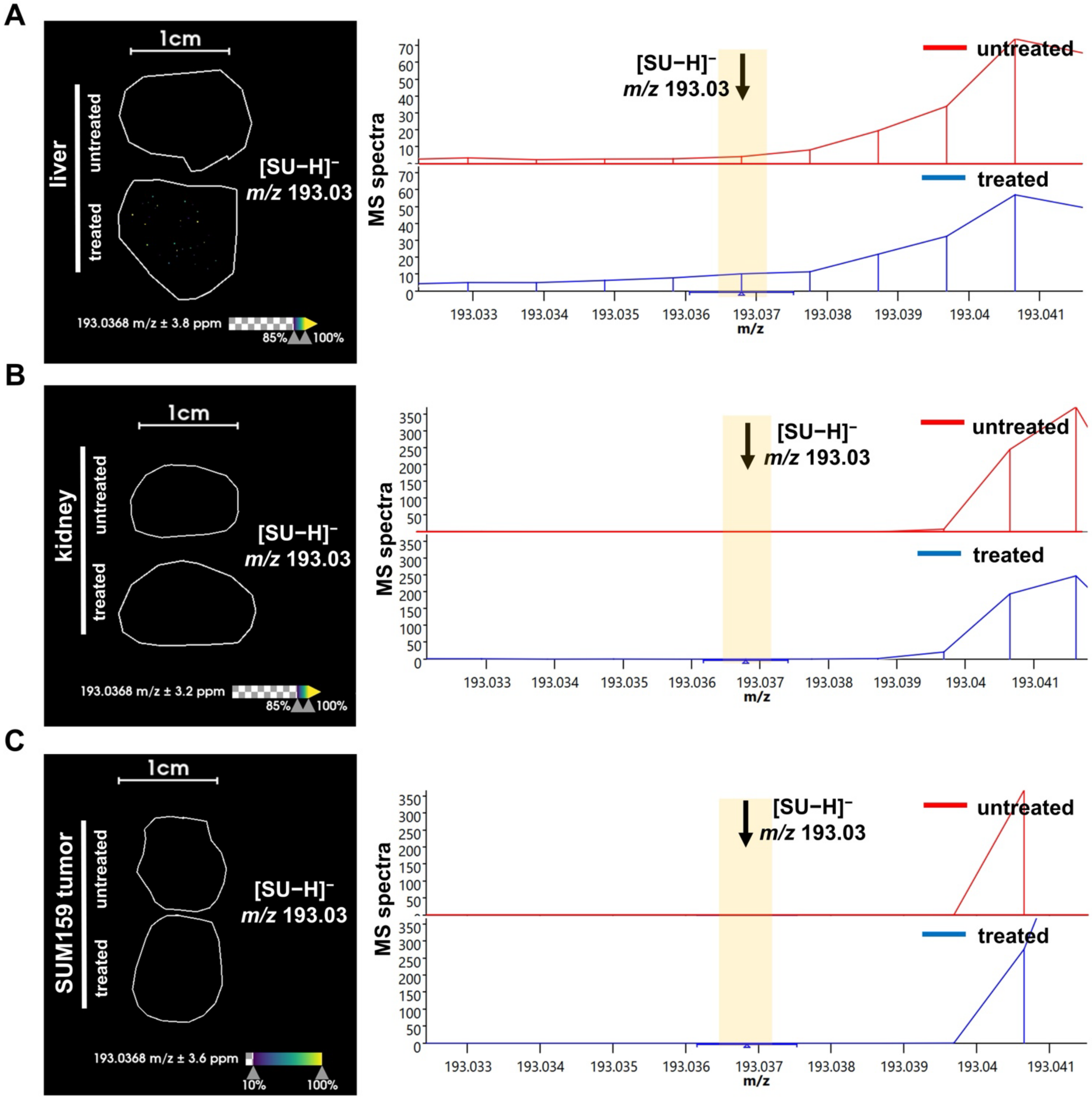

<u>Figure S12.</u> MALDI imaging of salicyluric acid (SU) distribution in tissue sections from aspirin treated versus untreated control mice. MALDI imaging and corresponding average spectra of SU distribution in representative (**A**) liver, (**B**) kidney, and (**C**) SUM159 tumor sections from 300 mM aspirin-treated (bottom, blue spectra) and untreated control (top, red spectra) mice. The data show that the $[M{-}H]^-$ ion of SU at *m/z* 193.03 Da was not detected in any tissues, neither in tissues from aspirin treated mice nor in those from untreated control mice. The MALDI images were acquired at 100 µm pixel size in negative ion mode using a timsTOF fleX MALDI-2 instrument with norharmane matrix and PAA as an additive.

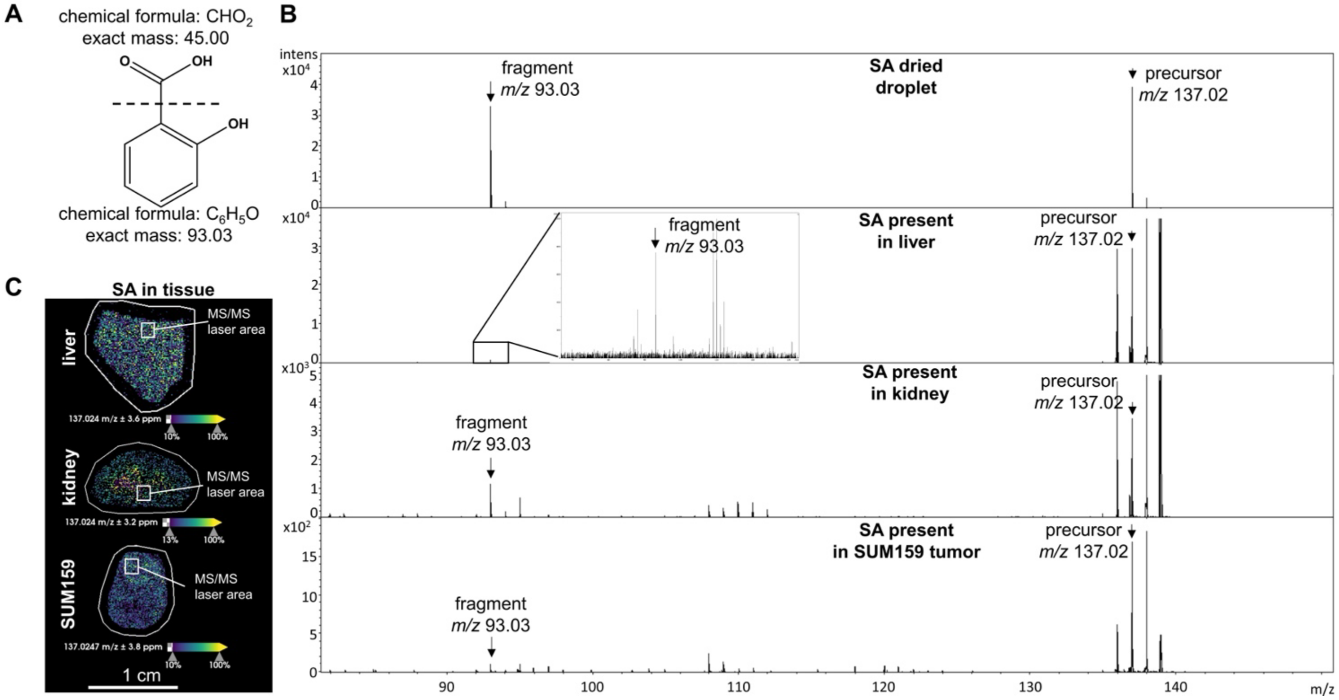

<u>Figure S13.</u> MS/MS spectra in negative ion mode for *m/z* 137.02, identified as SA. (**A**) The chemical structures of characteristic fragments are illustrated. (**B**) The precursor peak at *m/z* 137.02 Da and its fragment ion at *m/z* 93.03 Da are shown (top to bottom) from a dried SA droplet, and from liver, kidney, and SUM159 tumor sections. (**C**) The MS/MS laser area used for the analysis is highlighted with a white box.

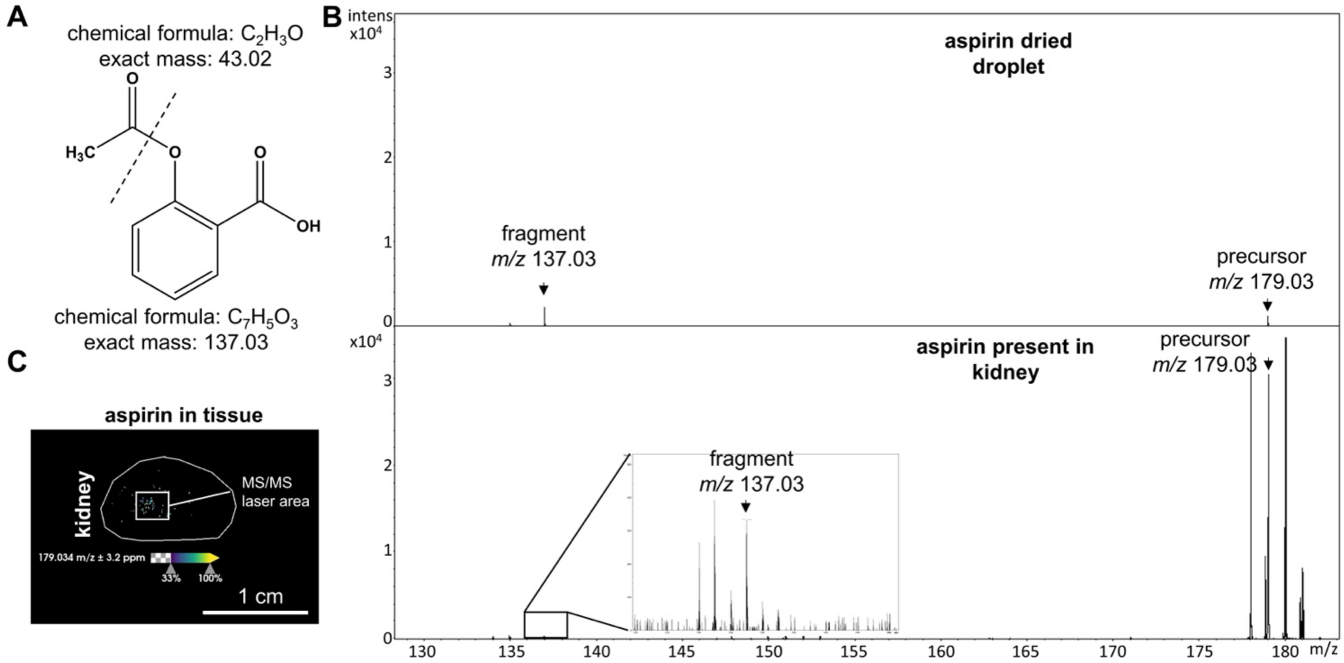

<u>Figure S14.</u> MS/MS spectra in negative ion mode for *m/z* 179.03, identified as aspirin. (**A**) The chemical structures of characteristic fragments are illustrated. (**B**) The precursor peak at *m/z* 179.03 Da and its fragment ion at *m/z* 137.03 Da are shown from a dried aspirin droplet (top) and in a kidney section (bottom). (**C**) The MS/MS laser area used for the analysis is highlighted with a white box.

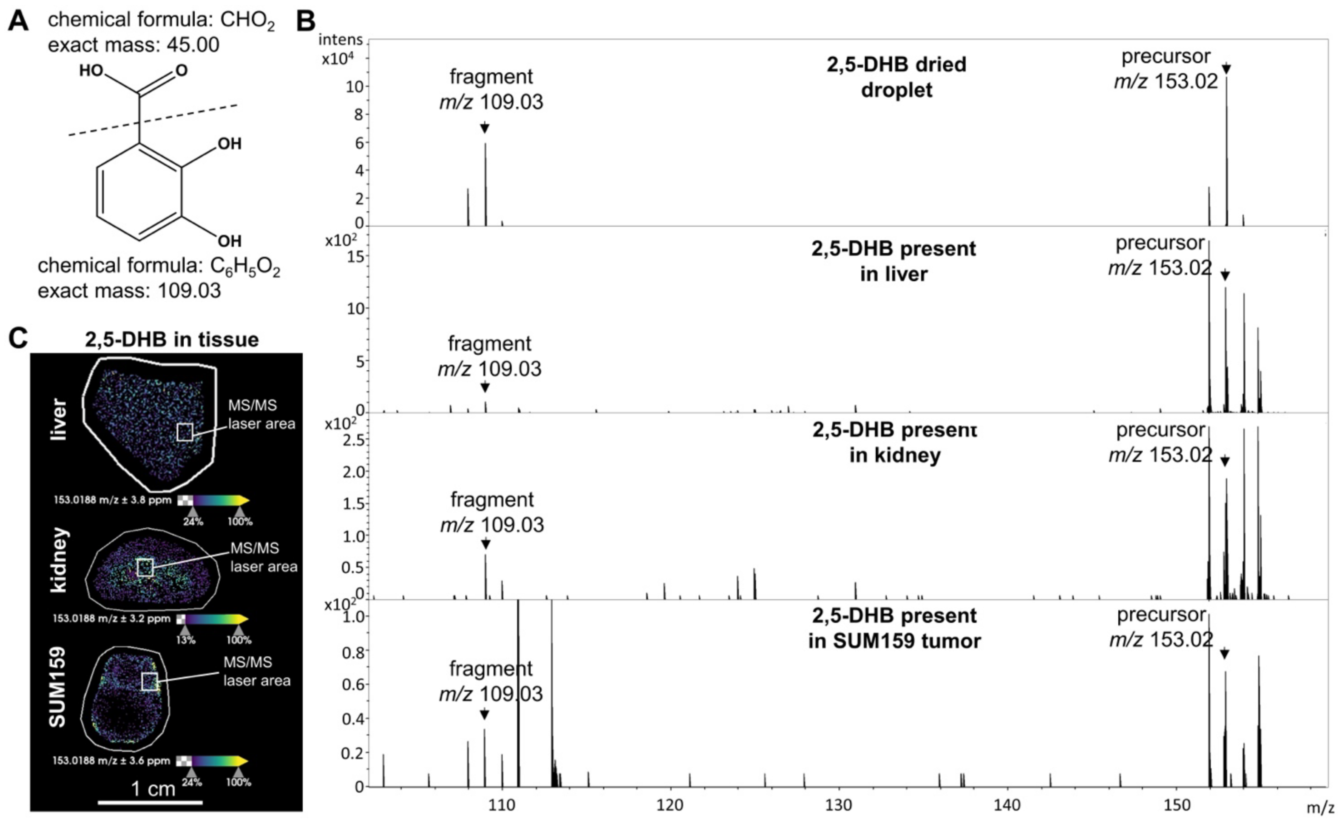

Figure S15. **MS/MS spectra in negative ion mode for *m/z* 153.02, identified as 2,5-DHB.** (**A**) The chemical structures of characteristic fragments are illustrated. (**B**) The precursor peak at *m/z* 153.02 Da and its fragment ion at *m/z* 109.03 Da are shown from a dried 2,5-DHB droplet, and from liver, kidney, and SUM159 tumor sections. (**C**) The MS/MS laser area used for the analysis is highlighted with a white box.

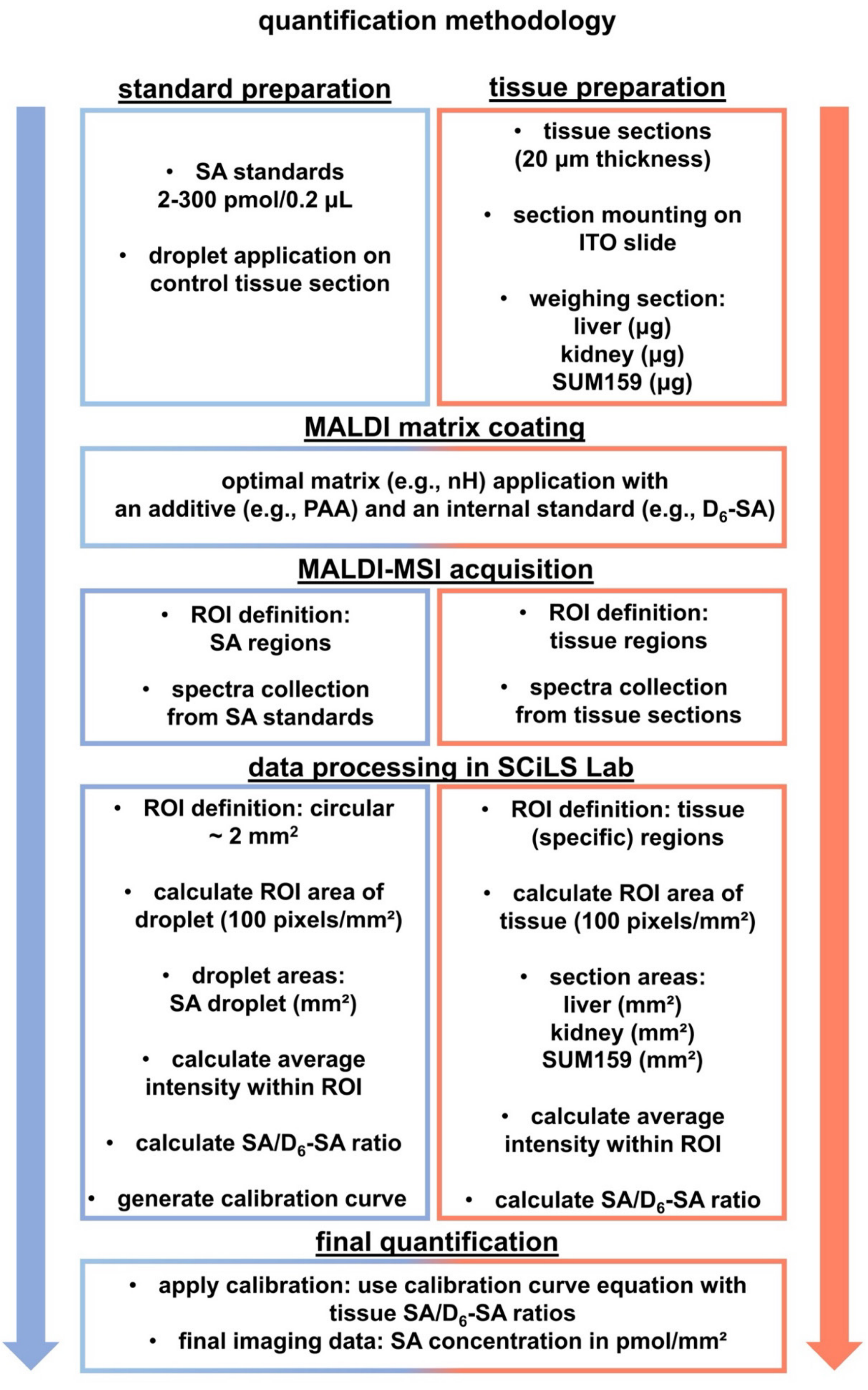

Figure S16. Comprehensive workflow map for SA quantification in tissue samples by QMALDI-MSI.

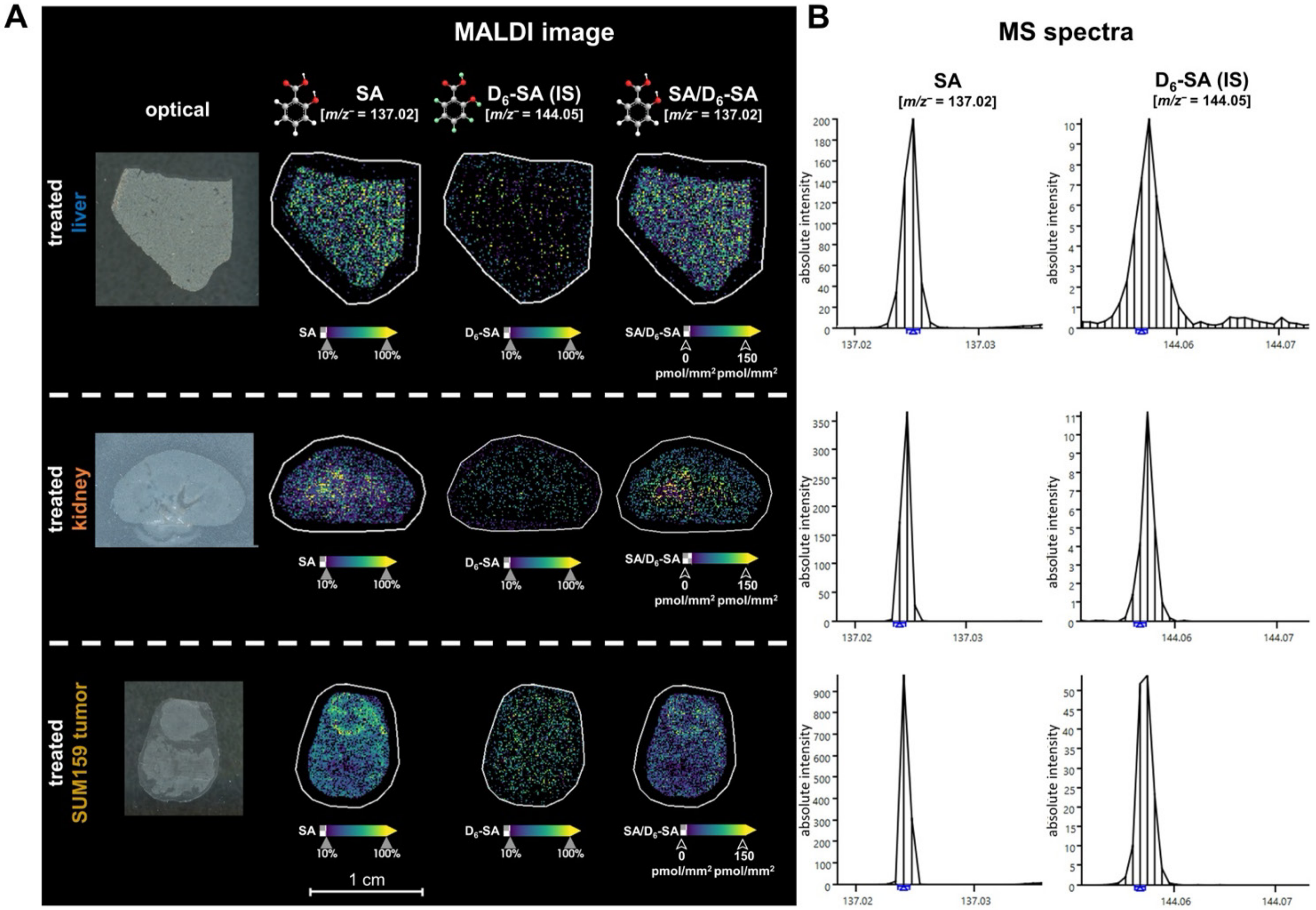

<u>Figure S17.</u> MALDI imaging analysis of sprayed on D_6-SA and tissue-contained SA in liver, kidney, and SUM159 tumor tissue sections. (**A**) Optical and MALDI images of uncorrected SA, D_6-SA, and normalized SA as SA-to-D_6-SA ratio were acquired and are shown from data analysis in SCiLS Lab software. (**B**) Representative mass spectra showing the detection of both sprayed on D_6-SA and tissue-contained SA in liver, kidney, and SUM159 tumor tissues. All spectral data were acquired and analyzed using SCiLS Lab software.

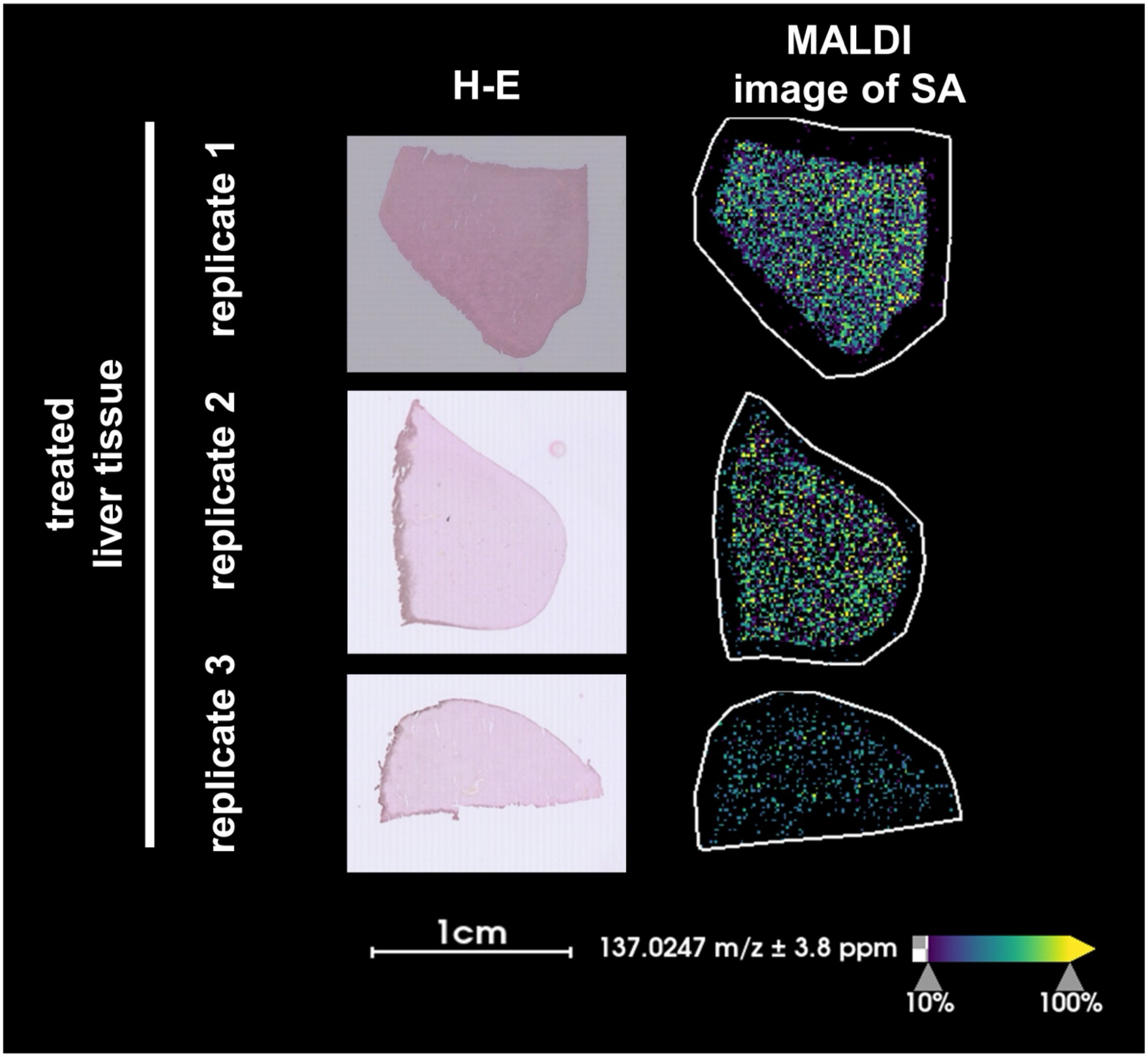

<u>Figure S18.</u> Microscopic images of liver sections stained with Hematoxylin-Eosin (H-E), alongside MALDI imaging. Microscopic images of H-E-stained liver sections from three biological replicates, accompanied by corresponding MALDI images showing the distribution of SA (*m/z* 137.02 Da, $[M{-}H]^-$) in liver tissue sections from aspirin treated mice. MALDI images were acquired using a timsTOF fleX MALDI-2 instrument with norharmane matrix and peracetic acid additive at 100 µm pixel size in negative ion mode.
